# Peptidoglycan Recycling in Gram-Positive Bacteria Is Crucial for Survival in Stationary Phase

**DOI:** 10.1128/mBio.00923-16

**Published:** 2016-10-11

**Authors:** Marina Borisova, Rosmarie Gaupp, Amanda Duckworth, Alexander Schneider, Désirée Dalügge, Maraike Mühleck, Denise Deubel, Sandra Unsleber, Wenqi Yu, Günther Muth, Markus Bischoff, Friedrich Götz, Christoph Mayer

**Affiliations:** a Department of Biology, Interfaculty Institute of Microbiology and Infection Medicine, University of Tübingen, Tübingen, Germany; b Institute of Medical Microbiology and Hygiene, University of Saarland, Homburg, Saar, Saarland, Germany

## Abstract

Peptidoglycan recycling is a metabolic process by which Gram-negative bacteria reutilize up to half of their cell wall within one generation during vegetative growth. Whether peptidoglycan recycling also occurs in Gram-positive bacteria has so far remained unclear. We show here that three Gram-positive model organisms, *Staphylococcus aureus*, *Bacillus subtilis*, and *Streptomyces coelicolor*, all recycle the sugar *N*-acetylmuramic acid (MurNAc) of their peptidoglycan during growth in rich medium. They possess MurNAc-6-phosphate (MurNAc-6P) etherase (MurQ in *E. coli*) enzymes, which are responsible for the intracellular conversion of MurNAc-6P to *N*-acetylglucosamine-6-phosphate and d-lactate. By applying mass spectrometry, we observed accumulation of MurNAc-6P in MurNAc-6P etherase deletion mutants but not in either the isogenic parental strains or complemented strains, suggesting that MurQ orthologs are required for the recycling of cell wall-derived MurNAc in these bacteria. Quantification of MurNAc-6P in Δ*murQ* cells of *S. aureus* and *B. subtilis* revealed small amounts during exponential growth phase (0.19 nmol and 0.03 nmol, respectively, per ml of cells at an optical density at 600 nm [OD_600_] of 1) but large amounts during transition (0.56 nmol and 0.52 nmol) and stationary (0.53 nmol and 1.36 nmol) phases. The addition of MurNAc to Δ*murQ* cultures greatly increased the levels of intracellular MurNAc-6P in all growth phases. The Δ*murQ* mutants of *S. aureus* and *B. subtilis* showed no growth deficiency in rich medium compared to the growth of the respective parental strains, but intriguingly, they had a severe survival disadvantage in late stationary phase. Thus, although peptidoglycan recycling is apparently not essential for the growth of Gram-positive bacteria, it provides a benefit for long-term survival.

## IMPORTANCE

The peptidoglycan of the bacterial cell wall is turned over steadily during growth. As peptidoglycan fragments were found in large amounts in spent medium of exponentially growing Gram-positive bacteria, their ability to recycle these fragments has been questioned. We conclusively showed recycling of the peptidoglycan component MurNAc in different Gram-positive model organisms and revealed that a MurNAc-6P etherase (MurQ or MurQ ortholog) enzyme is required in this process. We further demonstrated that recycling occurs predominantly during the transition to stationary phase in *S. aureus* and *B. subtilis*, explaining why peptidoglycan fragments are found in the medium during exponential growth. We quantified the intracellular accumulation of recycling products in MurNAc-6P etherase gene mutants, revealing that about 5% and 10% of the MurNAc of the cell wall per generation is recycled in *S. aureus* and *B. subtilis*, respectively. Importantly, we showed that MurNAc recycling and salvaging does not sustain growth in these bacteria but is used to enhance survival during late stationary phase.

## INTRODUCTION

Peptidoglycan (PGN) encases the bacterial cell, forming a huge, netlike, turgor-resisting and shape-maintaining envelope structure that is composed of glycan strands of two alternating β-1,4-linked sugars, *N*-acetylglucosamine (GlcNAc) and *N*-acetylmuramic acid (MurNAc), cross-linked by short peptides (1). The PGN is remarkably dynamic and is constantly remodeled, degraded, and rebuilt during bacterial growth and cell division (2, 3). As an inherent part of this process, a significant portion of the PGN is continuously excised from the cell wall by the activity of endogenous lytic enzymes (autolysins) and released into the medium in a process termed “turnover” (3–7). The reason for the continuous degradation of the PGN is still unclear, but apparently, a minimal set of autolytic enzymes is essential for bacterial growth, and PGN turnover may be intrinsically coupled with cell elongation and division (2, 5). Early studies showed that the Gram-negative bacterium *Escherichia coli* breaks down about half of its PGN within one generation during exponential growth (8, 9). Gram-negative bacteria possess a thin PGN layer embedded within an inner and an outer membrane, and thus, PGN turnover products are mostly retained within the periplasm, from where they are instantly recovered and salvaged (7, 8). Thus, only small amounts of PGN turnover products were found in the medium, while about 45% of the mature PGN was recovered in one generation in a process called cell wall or PGN recycling (8, 9). Uehara and Park later revealed that *E. coli* continuously recycles about 30% of newly synthesized septal PGN during cell division, and they calculated that about 60% of the PGN of the sidewall is recycled, taking into account that the PGN of the cell poles is basically inert (10). To quantify PGN turnover and recycling in *E. coli*, cell walls were radioactively prelabeled and the amounts of radioactive soluble products that were released into the medium or accumulated in recycling mutants were determined (8–10).

**FIG 1:**
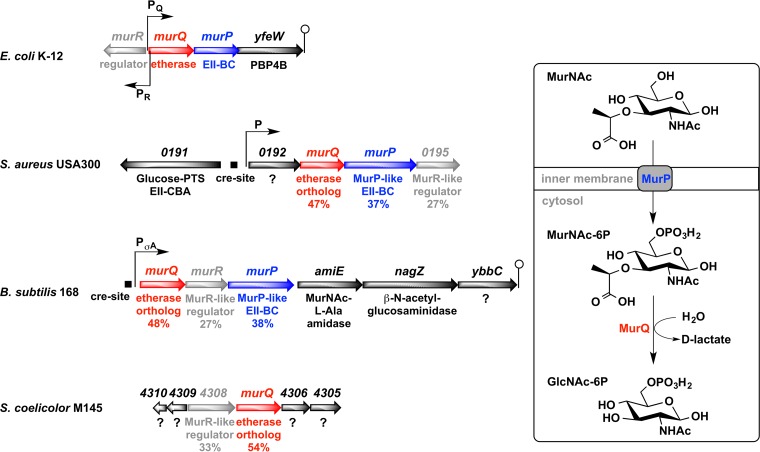
MurQ operon (MurNAc-recycling divergon) and MurNAc catabolic pathway in *E. coli* (top and right, respectively), and organization of chromosomal regions of *murQ* orthologs of the Gram-positive bacteria *S. aureus*, *B. subtilis*, and *S. coelicolor*. The schematic of the organization of the *E. coli* K-12 *murQ* operon genes includes the promoters for transcription of the *murQ* operon (P_q_) and *murR* (P_R_) and the terminator (lollipop), according to Jaeger and Mayer (20). *murQ* encodes MurNAc-6P etherase, *murP* encodes the MurNAc-specific phosphotransferase system (PTS) transporter EII-BC domain, and *yfeW* encodes the low-affinity penicillin binding protein 4B (PBP4B). Upstream from the *murQ* gene and divergently transcribed is the *murR* gene, a transcriptional repressor of the MurNAc recycling divergon. The schematic for *S. aureus* USA300 (NCBI Reference Sequence accession no. NC_007793.1) shows putative genes for MurNAc utilization, as well as the proteins they encode. *SAUSA300*_*0192* encodes a protein whose function is unknown, *SAUSA*_*0193* encodes an ortholog of MurQ, *SAUSA*_*0194* encodes an MurP-like PTS EII-BC domain protein, and *SAUSA*_*0195* encodes a MurR-like regulator. The schematic for *B. subtilis* 168 (NCBI Reference Sequence accession no. NC_000964.3) shows the putative promoter P_σA_ in front of the recycling cluster of 6 genes, including *murQ* (formerly *ybbI*), *murR* (formerly *ybbH*), *murP* (formerly *ybbF*), encoding the MurP EII-BC domain, *amiE* (formerly *ybbE*), encoding the MurNAc-l-alanine amidase AmiE, *nagZ* (formerly *ybbD*), encoding the *N*-acetylglucosaminidase NagZ, and *ybbC*, encoding a protein whose function is unknown. The schematic for *S. coelicolor* A3(2)/M145 (NCBI Reference Sequence accession no. NC_003888.3) shows a putative cluster of genes for MurNAc recycling that includes *SCO4308*, encoding a MurR-like regulator, and *SCO4307*, encoding an ortholog of MurQ, as well as two open reading frames encoding proteins whose functions are unknown, *SCO4305* and *SCO4306*. The amino acid sequence identities (%) of orthologous proteins relative to the sequences of *E. coli* MurQ, MurP, and MurR are shown. Catabolite-responsive elements (*cre* sites) were identified in the promoter regions upstream from the *murQ* genes of *B. subtilis* and *S. aureus*.

PGN recycling is not essential, at least under laboratory conditions, and why this pathway was kept on the chromosomes of most bacteria remained enigmatic (7). The PGN recycling metabolism of Gram-negative bacteria had attracted much attention due to a connection with AmpC-type β-lactamase induction (9) and, more recently, fosfomycin antibiotic resistance (11, 12). It is now reasonably well understood (for reviews, see references 3, 7, 13, and 14). In brief, the major cell wall-recycling products of Gram-negative bacteria are anhydromuropeptides (GlcNAc-β-1,6-anhydroMurNAc peptides) that are generated by the action of endogenous lytic transglycosylases and endopeptidases (autolysins), which in part are constituents of the huge cell wall synthetic complexes of elongation and division (2, 15). Anhydromuropeptides are actively transported into the cell by the AmpG permease and are further degraded in the cytoplasm by a mechanism that involves a set of dedicated recycling enzymes, mostly discovered and characterized in the seminal work of J. T. Park and coworkers (for reviews, see references 3 and 7). The recycling enzymes *N*-acetylglucosaminidase NagZ, l,d-carboxypeptidase LdcA, *N*-acetylmuramyl-l-alanine amidase AmpD, and anhydroMurNAc kinase AnmK process anhydromuropeptides in the cytoplasm, finally yielding MurNAc-6-phosphate (MurNAc-6P), besides other products. In addition, MurNAc-6P is the product of MurNAc uptake and concomitant phosphorylation by the specific phosphotransferase system (PTS) transporter MurP, allowing *E. coli* to grow on MurNAc as a sole source of carbon (16). A distinctive recycling enzyme that *E. coli* requires in order to catabolize MurNAc, as well as anhydroMurNAc, is the MurNAc-6P etherase MurQ (17, 18). This enzyme cleaves off the lactyl ether substituent from the phosphorylated form of MurNAc, yielding GlcNAc-6-phosphate (GlcNAc-6P) and d-lactate. The *murQ* operon of *E. coli* strain K-12 consists of three genes, encoding the MurNAc-6P etherase MurQ (17), the MurNAc transporter MurP (PTS EII-BC domain) (16), and a low-affinity penicillin binding protein named PBP4B (19) (Fig. 1). The transcriptional regulator MurR is transcribed divergently from the *murQ* operon and functions as a repressor in the absence of MurNAc-6P (20). Orthologs of *murQ* are missing in some Gram-negative bacteria, including *Pseudomonas* species (11), which instead use an alternative MurNAc-6P recycling route that bypasses the *de novo* PGN biosynthesis pathway (11, 12, 21).

It has long been assumed that large amounts of PGN fragments are shed from the cell wall of Gram-positive bacteria during vegetative growth and released into the growth medium (5, 22–24). However, whether these fragments are taken up and recycled in Gram-positive bacteria is currently unclear. Gram-positive bacteria have a thick PGN layer and lack an outer membrane, and hence, PGN turnover products are lost to the medium as they cannot be trapped in the periplasm as in Gram-negative bacteria. Applying continuous radioactive labeling and pulse-chase labeling approaches, the amount of PGN breakdown was measured by determining the radioactivity found in the growth medium relative to that remaining in the insoluble cell wall material. These studies came up with rather inconsistent rates of PGN release in Gram-positive bacteria during growth, ranging from no turnover to up to 50% turnover per generation (5, 22–25). The first reports revealed PGN turnover in the Gram-positive bacterium *Bacillus megaterium*, i.e., the release of PGN fragments into the growth medium (4), at a rate of 15 to 20% turnover per generation by measuring the loss of radiolabeled diaminopimelic acid from the prelabeled cell wall. Later, Mauck et al. calculated a rate of 30 to 50% PGN turnover per generation in *Bacillus subtilis* based on labeling studies using [^14^C]glucosamine and [^14^C]glutamine (22). *Staphylococcus aureus* strains were also described to turn over their cell wall at constant rates of up to 25% per generation (26) or about 15% per generation (27) during growth. Thus, the reported turnover rates differ to a large extent. Notably, Pooley emphasized that turnover rates determined with pulse-chase experiments often result in an overestimation of the amount of turnover, since there is a time lag between PGN synthesis and PGN degradation (25). He argued that the overall rate of turnover of the cell wall in *B. subtilis* is more likely about 8% per generation and is maintained by an approximately sixfold-higher rate of turnover of a small fraction, the old cell wall, while the major part, the newer wall, is initially resistant to turnover (25). Contradictory data, however, reported no or only very limited turnover in *B. megaterium* (28), as well as in *Streptococcus* bacteria (23). Recently, Boersma et al. confirmed that in the ovococcal *Streptococcus pneumoniae*, only minimal PGN turnover proceeds, indicated by the persistence of fluorescent d-amino acid label (29).

Still, it has not been investigated so far whether turnover fragments are recovered from the culture supernatant and recycled by Gram-positive bacteria (see reference 24 and references therein). We have previously identified putative PGN recycling genes on the chromosomes of Gram-positive bacteria and have characterized some of the encoded enzymes (24, 30). In particular, we recognized the general presence of *murQ* orthologs on the chromosomes of Gram-positive bacteria (11, 20), indicating that MurNAc recovery may occur in these organisms (Fig. 1). However, a clear proof of cell wall recycling during vegetative growth of Gram-positive bacteria is still missing, and the role of MurNAc-6P etherase enzymes in this process remains unclear. Thus, we constructed markerless MurNAc-6P etherase gene deletion mutants of the three Gram-positive model organisms *B. subtilis*, *S. aureus* and *Streptomyces coelicolor* and investigated the intracellular accumulation of MurNAc-6P in these strains during different growth phases by using mass spectrometry—hence, without the necessity to radioactively label the cell wall. Our study provides clear evidence for MurNAc recycling, i.e., the release of the sugar from the cell’s own peptidoglycan wall and its uptake/scavenging, occurring predominantly during the transition to stationary growth phase. Strikingly, PGN does not affect the growth rates of *S. aureus* and *B. subtilis* but is crucial for their survival during stationary phase.

## RESULTS

### Identification of putative MurNAc-recycling gene clusters in Gram-positive bacteria.

The organization of genomic *murQ* regions of selected Gram-positive bacteria, as well as of *E. coli*, and a schematic representation of the MurNAc catabolic pathway in *E. coli* are depicted in Fig. 1. On the chromosome of the *S. aureus* USA300 isolate FPR3757 (hereinafter referred to as USA300), we identified orthologs of *murQ* (*SAUSA300_0193*), *murP* (*SAUSA300_0194*), and *murR* (*SAUSA300_0195*) of *E. coli*, organized in a putative operon along with *SAUSA300*_*0192*, whose function is unknown. *SAUSA300*_*0193*–*0195* encode proteins with 47%, 37%, and 27% amino acid sequence identities to the respective *E. coli* proteins, based on analysis of the full-size proteins using the basic local alignment search tool (BLAST). A putative MurNAc-recycling operon was also identified on the genome of *B. subtilis* strain 168, containing the genes *murQ*, *murR*, and *murP* (formerly *ybbI*, *ybbH*, and *ybbF*, respectively), which encode proteins with amino acid sequence identities of 48%, 27%, and 38% to the respective *E. coli* enzymes. Downstream from *murQRP*, three further genes are located on the *B. subtilis* chromosome, *amiE* (formerly *ybbE*), *nagZ* (formerly *ybbD*), and *ybbC*, which encodes a protein of unknown function and has not been renamed. The first two genes encode the *N*-acetylmuramyl-l-alanine amidase AmiE and *N*-acetylglucosaminidase NagZ of *B. subtilis*, which were shown to be involved in the sequential extracellular degradation of muropeptides released during PGN turnover (30). On the genome of *S. coelicolor* strain A3(2)/M145, genes encoding an ortholog of the MurQ etherase (SCO4307; 54% amino acid identity to MurQ of *E. coli*) and a putative MurR-like regulator (SCO4308; 33% identity to MurR of *E. coli*) were identified by BLAST analysis; however, an ortholog of MurP is missing. These genes are organized in a putative operon along with the genes *SCO4305* and *SCO4306*, which encode proteins whose functions are unknown (Fig. 1).

As putative orthologs of the MurNAc-6P etherase MurQ of *E. coli* are present in all three Gram-positive bacteria and as *S. aureus* and *B. subtilis* also possess putative orthologs of the MurNAc-PTS transporter MurP of *E. coli*, we assumed that MurNAc can be catabolized and possibly recycled in these organisms according to the *E. coli* pathway depicted in Fig. 1.

### MurNAc-6P accumulation in Δ*murQ* mutants.

We constructed markerless in-frame deletion mutations of the respective MurNAc-6P etherase genes (referred to as Δ*murQ* mutants for all three species hereinafter) in *S. aureus* USA300, *B. subtilis* 168, and *S. coelicolor* strain M145 to investigate whether these Gram-positive bacteria recycle MurNAc derived from their own PGN, involving MurNAc-6P etherase. We expected that if recycling of the MurNAc content of their PGN occurred, Δ*murQ* cells would accumulate MurNAc-6P intracellularly. In addition, we generated an *S. aureus* USA300 deletion mutant lacking the entire putative *murQPR* operon (Δ*SAUSA*_*0192–0195*) and, in addition, a *B. subtilis* 168 Δ*murQRP* deletion mutant*.* Both of these mutants lack *murQ* and the genes encoding the putative MurNAc PTS transporters. Thus, these mutants should not be able to take up MurNAc released from the PGN wall and therefore should not accumulate MurNAc-6P. Gene deletions on the chromosome were confirmed by PCR (see Fig. S1 in the supplemental material) and sequencing.

**FIG 2:**
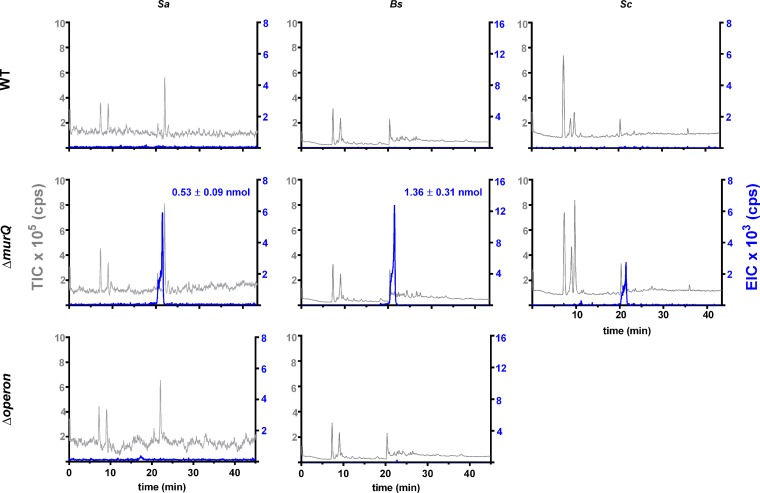
Accumulation of MurNAc-6P in *ΔmurQ* mutants of Gram-positive bacteria but not in the parental strains. *S. aureus* (*Sa*), *B. subtilis* (*Bs*), and *S. coelicolor* (*Sc*) wild-type strains (WT) and Δ*murQ* mutants, as well as the *S. aureus* Δ*SAUSA*_*0192–0195* and *B. subtilis* Δ*murQRP* mutants (Δoperon), were cultured in LB medium for 24 h. Acetone extracts of cytosolic fractions were analyzed by LC-MS in negative-ion mode. Mass spectra of MurNAc-6P in the investigated samples are presented with the total-ion chromatograms (TIC) (×10^5^ counts per s [cps]) in gray and the extracted-ion chromatograms (EIC) (×10^3^ cps) (*m*/*z*^−1^ = 372.07 and retention time on the HPLC column of 21 min) in blue.

At first, we investigated the intracellular accumulation of MurNAc-6P in cells grown for 24 h in nutrient-rich medium (LB medium). Cytosolic fractions were obtained by disrupting the cells with glass beads and were subsequently extracted with acetone prior to analysis by liquid chromatography-mass spectrometry (LC-MS). In the extracts of all Δ*murQ* mutants of *S. aureus*, *B. subtilis*, and *S. coelicolor*, we detected MurNAc-6P (Fig. 2), which was identified based on identity with a standard according to retention time during high-performance liquid chromatography (HPLC) separation and on the exact mass (retention time on the HPLC column of 21 min and *m*/*z*^−1^ of 372.07) (see Fig. S2 in the supplemental material). In contrast, MurNAc-6P was not detected in any corresponding parental strain and, notably, not in *S. aureus* ΔmurQPR and *B. subtilis* ΔmurQRP mutant cells either, indicating that the accumulation of MurNAc-6P, and hence recycling, relies on both a functional MurQ etherase and the presence of the putative MurNAc PTS transporter (Fig. 2). The absence of MurNAc-6P accumulation in these operon mutants further indicates that the MurQP pathway is the only route for MurNAc recycling via MurNAc-6P in these organisms.

### Δ*murQ* mutants have no growth defects, and the addition of MurNAc does not enhance growth in rich medium.

**FIG 3:**
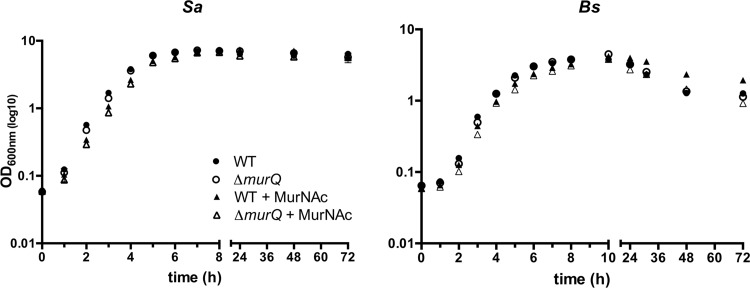
Growth of *S. aureus* and *B. subtilis* wild-type strains and Δ*murQ* mutants in rich medium with or without MurNAc. *S. aureus* (*Sa*) and *B. subtilis* (*Bs*) wild-type strains (WT, solid symbols) and the respective mutants (Δ*murQ*, open symbols) were grown in LB medium in the absence (circles) or presence (triangles) of 0.2% MurNAc. Bacterial growth was monitored by measuring optical density at 600 nm and is presented as the mean values ± standard errors of the means (SEM) in log_10_ scale.

We asked whether impairment of PGN recycling and the accumulation of MurNAc-6P would affect growth. Therefore, we monitored the optical density at 600 nm (OD_600_) during growth of wild-type and *ΔmurQ* strains of the unicellularly growing Gram-positive bacteria *S. aureus* and *B. subtilis* in LB medium. Surprisingly, the mutant strains displayed no growth deficiencies compared to the growth of the parental strains, even during incubation in stationary phase for 72 h (Fig. 3). The growth of *S. coelicolor* by apical tip extension results in multicellular mycelial pellets composed of stationary (old compartments) and actively growing (tip compartments) mycelium. Since this precludes the separation of distinct growth phases, *S. coelicolor* was excluded from these studies.

We further asked whether MurNAc added to the medium would support growth. Therefore, 0.2% MurNAc was added to the LB medium and the growth of wild-type and *ΔmurQ* strains of *S. aureus* and *B. subtilis* was followed during prolonged culturing by measuring the OD_600_. Surprisingly, externally supplied MurNAc did not support the growth of either strain but, rather, had a weak repressive effect on growth during exponential phase. Interestingly, in late stationary phase, wild-type *B. subtilis* cells yielded a higher OD_600_ in the presence of MurNAc. In contrast, wild-type *S. aureus* cells had no late-stationary-phase growth advantage in the presence of MurNAc.

### Growth-phase-dependent accumulation of MurNAc-6P.

We wondered in which phases of vegetative growth PGN recycling occurs. Therefore, we determined the amount of MurNAc-6P that accumulated in the *murQ* mutants in different growth phases in *S. aureus* and *B. subtilis*. The levels of MurNAc-6P accumulation were examined in extracts of the cytosolic fractions of Δ*murQ* mutants and parental strains (wild type) of *S. aureus* and *B. subtilis* in exponential growth phase (OD_600_ of 3 and 2, respectively), transition phase (OD_600_ of 7.5 and 4), and stationary phase (OD_600_ of 6.5 and 3) (see Fig. S3 in the supplemental material). Quantification of the accumulation product was achieved by comparison to the MurNAc-6P standard, which was generated from MurNAc in an enzymatic synthesis reaction with MurNAc kinase from *Clostridium acetobutylicum* (31) and subsequently purified by HPLC as described elsewhere (S. Unsleber, M. Borisova, and C. Mayer, unpublished data). Dilution series of the MurNAc-6P standard were analyzed by LC-MS, and the area under the curve was determined for the product peaks (see Fig. S2 in the supplemental material) and used to quantify the amounts of recycling product that accumulated in Δ*murQ* mutants during the growth phases analyzed.

MurNAc-6P was absent from all wild-type samples in the three growth phases tested (Fig. 2; see also Fig. S3 in the supplemental material). The *S. aureus* Δ*murQ* mutant accumulated MurNAc-6P only in small amounts during exponential phase (for cell cultures with an OD_600_ of 1 [OD1 cells], the amounts of MurNAc-6P were 0.19 ± 0.02 nmol/ml of OD1 cells [mean ± standard error of the mean); however, the amounts increased threefold in transition phase (0.56 ± 0.1 nmol) (see Fig. S3) and stationary phase (0.53 ± 0.09 nmol), respectively (Fig. 2). Similarly, in *B. subtilis*, small amounts of MurNAc-6P were detected in the Δ*mur*Q samples during exponential phase (0.030 ± 0.005 nmol/ml of OD1 cells), the amounts detected increased significantly in transition phase (0.52 ± 0.07 nmol) (see Fig. S3), and MurNAc-6P reached the maximum intracellular amounts (1.36 ± 0.31 nmol) in stationary phase after 24 h of culturing (Fig. 2).

We then complemented the Δ*murQ* mutants of *S. aureus* and *B. subtilis* and studied the effect on the accumulation of MurNAc-6P. To complement the MurQ ortholog in *S. aureus*, the Δ*murQ* mutant was transformed with pRB474 constitutively expressing the MurQ ortholog from *S. aureus* (pRB474-*murQ*) or with the empty pRB474 plasmid as a control. By LC-MS analysis, we observed a 65% reduction in the amount of MurNAc-6P when the *S. aureus* MurQ ortholog was expressed in *trans* (see Fig. S4 in the supplemental material). For complementation of MurQ in *B. subtilis*, either the vector pX-*murQ* or the empty vector pX (control) was integrated into the *amyE* sites in the chromosome of the *B. subtilis* Δ*murQ* mutant. Enzyme expression was induced with 0.3% xylose, since the expression of MurQ in pX-*murQ* is under the control of a xylose-inducible promoter. MurQ complementation in the *B. subtilis* recycling mutant resulted in a complete disappearance of the intracellular MurNAc-6P that accumulated in the Δ*murQ* mutant (see Fig. S4).

To relate our data obtained with *S. aureus* and *B. subtilis* to the recycling data for a Gram-negative bacterium, we measured MurNAc-6P accumulation in *E. coli* wild-type and Δ*murQ* cells. It has been reported that MurNAc-6P accumulates in *E. coli* murQ (strain TJ2e) mutant cells grown in the absence and presence of external MurNAc as assayed by thin-layer chromatography and radiodetection (17, 18). Previously, *E. coli* MurNAc-6P was quantified only in stationary phase. Here, we detected already in mid-exponential phase large amounts of MurNAc-6P in the *E. coli* Δ*murQ* mutant (1.06 nmol) that remained high in the transition growth phase (0.99 nmol), whereas in the parental strain, no MurNAc-6P was detected (see Fig. S3 in the supplemental material).

Thus, the accumulation of MurNAc-6P in *E. coli* was already high in exponential phase. In contrast, we obtained only minimal amounts of MurNAc-6P from exponentially grown *ΔmurQ* cells of Gram-positive bacteria, particularly in *B. subtilis*, but much larger amounts from cells in transition and stationary phases. We aimed to clarify whether the apparent delay of PGN recycling in Gram-positive bacteria is due to a low activity or abundance of MurQ and MurP orthologs or to the small amounts of MurNAc provided from the breakdown of cell wall turnover products. Therefore, we added MurNAc (0.02%) to the culture medium and determined the intracellular concentrations of MurNAc-6P in *S. aureus* and *B. subtilis* wild-type and *ΔmurQ* cells at different growth phases (Fig. 3; see also Fig. S5 in the supplemental material). MurNAc-6P was generally absent in the cytosolic fractions of wild-type cells grown in LB medium supplemented with 0.02% MurNAc, except for small amounts measured in the *S. aureus* cells after 24 h of growth (see Fig. S5). However, in *S. aureus* ΔmurQ, the intracellular concentrations of MurNAc-6P in the presence of MurNAc increased in exponential phase to 2.33 ± 0.32 nmol/ml of OD1 cells and in transition phase to 6.21 ± 0.66 nmol/ml of OD1 cells, and they reached a maximum in stationary growth phase of 11.56 ± 0.39 nmol/ml of OD1 cells (see Fig. S5). Thus, *S. aureus* cells tolerated the very high intracellular concentrations of MurNAc-6P without showing any obvious growth defect, even with 0.2% exogenous MurNAc (Fig. 3). In *B. subtilis* ΔmurQ cells, the addition of MurNAc caused large increases in the intracellular MurNAc-6P levels, particularly in exponentially growing cells. When MurNAc was added to the medium, MurNAc-6P concentrations of 1.05 ± 0.05 nmol were determined in exponential phase. During transition phase, adding MurNAc to the medium caused the levels of MurNAc-6P to increase to 5.21 ± 0.73 nmol/ml of OD1 cells, and adding MurNAc in stationary phase caused the levels to increase to 3.11 ± 0.13 nmol/ml OD1 cells. Surprisingly, the levels of MurNAc-6P dropped from transition to stationary phase in *B. subtilis* ΔmurQ cells grown in LB with MurNAc (see Fig. S5). Reevaluation of the MS data revealed that the reduction of the amount of MurNAc-6P in stationary-phase cells correlated with an increase in MurNAc. This is most likely caused by an unknown phosphatase in *B. subtilis* that is responsible for dephosphorylation of MurNAc-6P, thus yielding MurNAc (data not shown).

### Cell wall recycling is crucial for survival in stationary phase.

**FIG 4:**
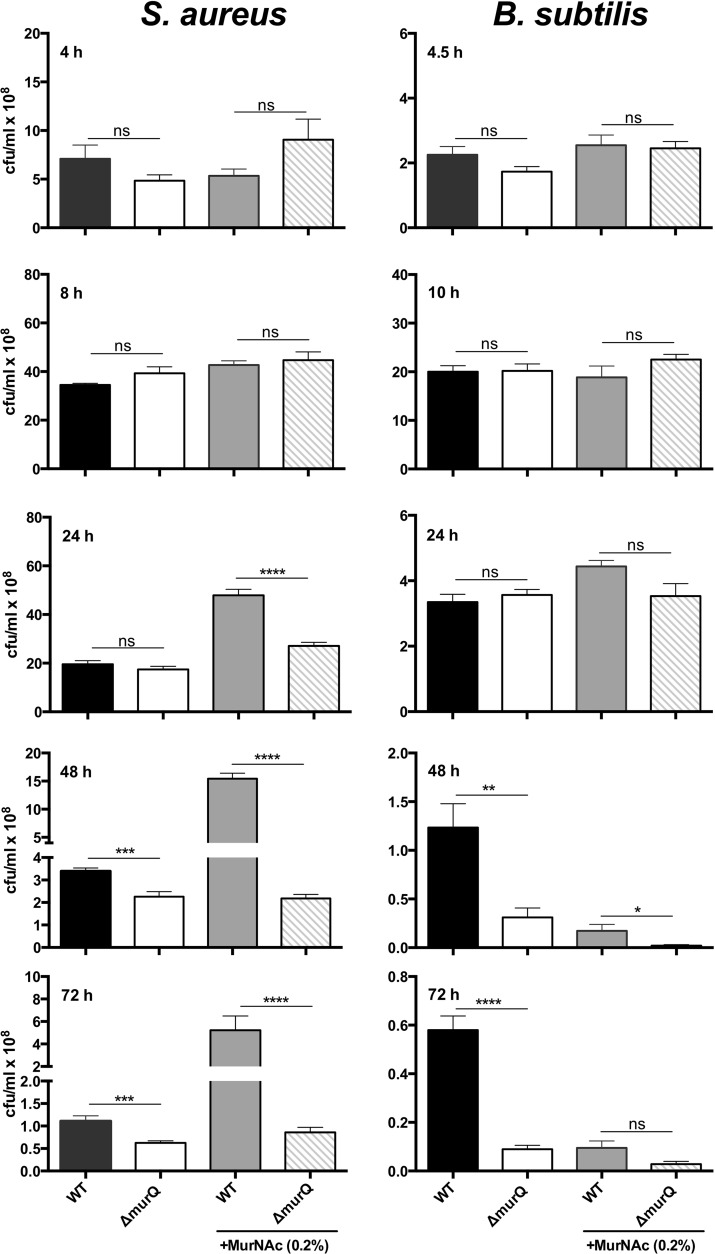
Determination of viable cell titers of *S. aureus* and *B. subtilis* wild-type strains (WT) and their Δ*murQ* mutants grown in LB medium with or without MurNAc. Wild-type and Δ*murQ* mutant cells of *S. aureus* and *B. subtilis* were grown in LB medium in the absence (left two bars in each graph) or presence of 0.2% MurNAc. Bacterial cultures were diluted appropriately in 0.9% NaCl solution in 96-well plates and plated on LB agar. Viable cells were determined by counting CFU/ml (×10^8^) at mid-exponential (4 h for *S. aureus* and 4.5 h for *B. subtilis*), transition (8 h and 10 h, respectively), and stationary (24 h, 48 h, and 72 h) phases. Data are presented as the mean values ± SEM from three independent biological replicates and were analyzed for statistical significance with the nonpaired *t* test. A *P* value of <0.05 was determined as statistically significant (*, *P* ≤ 0.05; **, *P* ≤ 0.01; ***, *P* ≤ 0.001; ****, *P* ≤ 0.0001; ns, nonsignificant).

Although large amounts of MurNAc-6P accumulate in Δ*murQ* cells of *S. aureus* and *B. subtilis* grown in LB and even more in the presence of MurNAc, the growth rates determined by optical density measurements were more or less identical for wild-type and mutant cells for at least 24 h of growth (Fig. 3). However, in late stationary phase in LB medium supplemented with 0.2% MurNAc, *B. subtilis* wild-type cultures remained at a higher OD (Fig. 3). We supposed that, although the impairment of recovery of the MurNAc of the peptidoglycan wall had no effect on growth, it might still affect cell survival. To investigate whether MurNAc recycling provides a survival benefit, we analyzed the viability of cells by determining the CFU counts (CFU/ml) (Fig. 4; see also Table S3 in the supplemental material). Similar cell numbers were determined for wild-type and Δ*murQ* strains grown to exponential phase and transition phase in medium with and without added MurNAc. Intriguingly, in stationary phase (24 h of culturing), only the wild-type *S. aureus* cell cultures supplemented with exogenous MurNAc maintained high cell numbers; in all other cases, the CFU counts dropped dramatically. This is not a growth but a survival effect, since the cell numbers did not increase between 8 h and 24 h of incubation of wild-type *S. aureus* cell cultures in medium with MurNAc but did remain at a high level, whereas cell numbers of the Δ*murQ* strain dropped by twofold. Notably, prolonged incubation of *S. aureus* cells in medium with MurNAc for 48 h and 72 h showed that wild-type cell cultures maintained seven- and sixfold higher cell numbers (CFU/ml), respectively, than did mutant cell cultures. A slight but significant survival advantage of *S. aureus* wild-type cells compared to the survival of mutant cells was also observed upon culturing for 48 h and 72 h in LB medium without MurNAc. Thus, prolonged incubation in the culture medium revealed a defect in the survival of the MurNAc-recycling-mutant cells compared to the survival of wild-type cells (Fig. 4; see also Table S3).

Similar data were obtained for *B. subtilis*. We observed no significant differences in the CFU counts of wild-type and mutant cell cultures within the first 24 h of growth in LB either in the absence or presence of MurNAc. However, the viability of *murQ* mutant cell cultures decreased dramatically during prolonged incubation in stationary phase (48 h and 72 h). In the presence of MurNAc, surprisingly, a dramatic decrease of viability was observed, possibly due to cell lysis at the onset of sporulation. Notably, this effect was even enhanced in the recycling mutant (Fig. 4; see also Table S3).

## DISCUSSION

We show here that three Gram-positive bacteria, *S. aureus*, *B. subtilis*, and *S. coelicolor*, recycle the MurNAc contents of their PGN cell wall during vegetative growth in rich medium, which requires the orthologs of the MurQ enzyme of *E. coli*. Thereby, we provide for the first time direct evidence for PGN recycling in Gram-positive bacteria and proof of MurQ function in these organisms. Since these differently growing species, the elongating rod-shaped *B. subtilis*, the orthogonally dividing coccoidal *S. aureus*, and the apically growing filamentous *S. coelicolor*, all recycle the MurNAc of their PGN, this pathway is presumably a general feature of Gram-positive bacteria*.* Indeed, orthologs of *murQ*, initially identified and related to PGN recycling in the Gram-negative model bacterium *E. coli* (17, 18), are found on the chromosomes of nearly all Gram-positive bacteria (an exception is *Streptococcus* species). We directly measured the recycling product MurNAc-6P that accumulated in the *murQ* mutants by applying a mass spectrometry-based method, which avoids radioactive prelabeling of cell walls and the subsequent measurement of the radioactive fragments released but still allows sensitive detection and quantification of MurNAc-6P in cell extracts within a large concentration range of 2.5 to 1,250 µM. Since the wild-type strains, as well as complemented mutants, contained no MurNAc-6P or greatly reduced amounts, respectively, it is evident that the MurQ etherases are required for MurNAc recycling. The possibility that MurNAc-6P accumulated in *ΔmurQ* mutants due to the presence of MurNAc in the growth medium could be excluded because MurNAc is absent from the LB culture medium (24, 32; data not shown). Furthermore, MurNAc-6P accumulation was not detected in strains of *S. aureus* and *B. subtilis* that were defective in both MurQ and the putative MurNAc transporter, indicating that the MurP MurNAc phosphotransferase transporters are the only uptake systems for MurNAc recycling in these organisms.

We determined the levels of MurNAc-6P that accumulated in *murQ* mutants of *S. aureus* and *B. subtilis* in different growth phases. The amounts of MurNAc-6P are presented as nanomoles per milliliter of OD1 cells. Assuming that 1 ml of OD1 cells contains a cell volume of about 1 µl, a value of 1 nmol would equate roughly to an intracellular accumulation of 1 mM of MurNAc-6P. We show here that MurNAc recycling is low during exponential growth and mainly occurs during transition and stationary phases in *S. aureus* and *B. subtilis*. These results largely explain why PGN turnover products are found in abundance in the culture medium of exponentially growing Gram-positive bacteria (see reference 24 and references therein). In Gram-positive bacteria, PGN recycling occurs not synchronous with turnover, as in *E. coli* (2), but after a delay. Turnover of the Gram-positive cell wall occurs by a so-called inside-to-outside growth mechanism (33). The PGN is first assembled near the plasma membrane and is then gradually moved to the outside, where it becomes stretched and more susceptible to the activity of autolysins. The small amounts of MurNAc-6P in exponential-phase cells, particularly in the *B. subtilis* Δ*murQ* mutant, can be explained by the delayed release of MurNAc from the PGN wall due to the slow autolytic processing of larger PGN turnover fragments. It is likely that MurNAc is released more slowly from the very long PGN chains present in *B. subtilis* than from the rather short PGN chains of *S. aureus* (34).

MurNAc recycling does not sustain the growth of *S. aureus* and *B. subtilis*; instead, it appears to be of major relevance for their survival capacity when growth is stalled during nutrient limitation at the onset of stationary phase and recycling may be used to overcome a shortage of nutrients and precursors for cell (wall) differentiation. Our results are in agreement with those of previous studies indicating that the transcription of *B. subtilis murQ* occurs mainly during the transition to stationary phase (35). Moreover, these results are consistent with previous findings showing that the expression of the recycling *N*-acetylglucosaminidase NagZ of *B. subtilis* is low during exponential phase but highly elevated in stationary phase (30). The enzyme releases GlcNAc from the PGN, and it is likely that besides MurNAc, the GlcNAc part of the PGN is also recycled; however, this would require uptake by a different transport system.

When MurNAc was added to the medium, the accumulation of MurNAc-6P increased greatly, indicating that the uptake and recycling of MurNAc is limited by the availability of the sugar. As mentioned above, the release of MurNAc depends on autolytic processing of PGN, which is growth phase-dependently regulated. In the proximity of *murQ* and its orthologs on the genomes of the bacteria studied, transcriptional regulator proteins were identified that, similar to the case for *E. coli* (20), may likely accelerate the transcription of the MurNAc transporter and MurQ in the presence of MurNAc-6P. Interestingly, in stationary phase, large amounts of MurNAc and MurNAc-6P were found in the cytosolic fractions of *B. subtilis* Δ*murQ* mutant cells grown in LB medium supplemented with MurNAc, which might be due to dephosphorylation of MurNAc-6P at high concentrations by a putative phosphatase. Notably, an enzyme with identical function was recently proposed in a *Pseudomonas* species (13).

To relate our quantitative data on MurNAc-6P accumulation in *S. aureus* and *B. subtilis* to its accumulation in the Gram-negative bacterium *E. coli*, we reexamined and quantified the MurNAc-6P accumulation in the *murQ* mutant of *E. coli* using our mass spectrometry-based method. In *E. coli* murQ mutant cells, the maximum accumulation of MurNAc-6P occurred already in mid-exponential growth phase and reached an intracellular concentration of c. 1 mM (1 nmol/ml of OD1 cells); Wientjes and colleagues reported that the PGN of one *E. coli* cell contains 3.5 × 10^6^ molecules of diaminopimelic acid (36), and presumably, similar numbers of MurNAc molecules are present. As 1 ml of a cell culture with an OD_600_ of 1 (OD1 cells) in exponential phase amounts to 5 × 10^8^ to 1 × 10^9^ cells, it can be calculated that one *E. coli* cell contains 1.7 × 10^15^ to 3.5 × 10^15^ molecules of MurNAc. Thus, a recycling rate of 45% per generation would lead to a MurNAc-6P accumulation of about 1.3 to 2.6 mM. The value of about 1 mM obtained in exponentially growing *E. coli* cells in this study is a little lower than the expected concentration range. Interestingly, the amounts of the recycling product MurNAc-6P determined in *E. coli* cells during mid-log phase and in the examined Gram-positive bacteria during transition to stationary phase were similar. This indicates that, compared to the rates of PGN synthesis, the turnover and recycling of PGN appear to be quite similar in both groups of bacteria. However, related to the thickness of the Gram-positive cell wall, with approximately 5- to 10-fold-greater PGN contents than in *E. coli*, it can be estimated that only about 5 to 10% of the PGN is recycled in *S. aureus* and *B. subtilis* per generation. These values are in the range of the 8% recycling reported for *B. subtilis* by Pooley (25) but are lower than the rates of turnover reported elsewhere (22, 26, 27).

The most intriguing finding of our study was that PGN sugar recycling affects survival fitness. Apparently, the cell wall sugar MurNAc does not serve primarily as an energy source but is preferentially utilized for cell wall synthesis. The same presumably holds for GlcNAc, as the MurNAc and GlcNAc catabolic pathways merge. Amino sugar utilization and its regulation were studied in *B. subtilis* and in *S. aureus* (37) and were compared with those of *E. coli* in a recent review (38). These studies revealed that *S. aureus* and *B. subtilis* use GlcNAc preferentially for cell wall synthesis; in *B. subtilis*, about 95% of GlcNAc from the medium is usually incorporated into PGN. Our findings are consistent with these observations. Both cell wall sugars, GlcNAc and MurNAc, are presumably used in *S. aureus* and *B. subtilis* for cell wall synthesis, at least during growth in rich medium. Combined with the data presented here, one could speculate that in Gram-positive bacteria, MurNAc recycled from the PGN, as well as that salvaged from the medium, is not used to gain energy or for growth but might be utilized to stabilize the cell wall, and thus, it increases survival during prolonged starvation. Although PGN recycling is not essential, a huge set of recycling genes are maintained in almost all bacterial genomes, suggesting that the pathway must provide a benefit for bacterial cells. We show here that PGN recycling is crucial for the survival of Gram-positive bacteria during stationary phase.

## MATERIALS AND METHODS

### Chemicals, enzymes, and oligonucleotides.

Enzymes for DNA restriction and for cloning were obtained from New England Biolabs (Ipswich, MA) or Thermo Fischer Scientific (Schwerte, Germany). The Gene JET plasmid miniprep kit, PCR purification kit, and Gene Ruler 1-kb marker were from Thermo Fisher Scientific and Qiagen (Venlo, Netherlands). *N*-Acetylmuramic acid (MurNAc) was from Bachem (Bubendorf, Switzerland), and the DNA dye NonTox was from Applichem (Darmstadt, Germany). Oligonucleotides were purchased from MWG Eurofins (Ebersberg, Germany) and are listed in Table S1 in the supplemental material.

### Bacterial strains, growth conditions, and construction of mutants and plasmids.

The plasmids and bacterial strains used in this study are shown in Table S2 in the supplemental material. The construction of mutant strains and plasmids is described in Text S1 in the supplemental material. *Escherichia coli* K-12, *Bacillus subtilis* 168, and *Staphylococcus aureus* USA300 were cultured aerobically in lysogeny broth (LB; 5 g/liter yeast extract, 10 g/liter tryptone, 10 g/liter NaCl) at 37°C and under continuous shaking at 160 rpm or on solid LB supplemented with 1.5% agar. *B. subtilis* and *S. aureus* overnight cultures (~16 h) were used to inoculate fresh LB medium to yield an initial optical density at 600 nm (OD_600_) of 0.05 for growth studies or the determination of intracellular accumulation of *N*-acetylmuramic acid-6 phosphate (MurNAc-6P) at different growth phases. Tryptic soy broth (TSB; Oxoid) was used to generate the markerless *S. aureus* Δ*murQ* mutant and for complementation experiments. *S. coelicolor* M145 was grown in LB medium with constant shaking (180 rpm) at 30°C. Antibiotics were used, when appropriate, at the following concentrations: ampicillin (100 µg/ml) for *E. coli*; chloramphenicol (5 µg/ml) and erythromycin and tetracycline (10 µg/ml) for *B. subtilis*; erythromycin (5 µg/ml) and chloramphenicol (10 µg/ml) for *S. aureus*; and apramycin (50 µg/ml) for *S. coelicolor*. For induction of the P*xylA′* promoter in *B. subtilis*, 0.3% xylose was added to the LB growth medium.

### Generation of cytosol fractions.

Overnight cultures of *S. aureus*, *B. subtilis*, and *E. coli*, including both the wild types and the respective Δ*murQ* mutants, were used to inoculate LB medium, yielding an initial OD_600_ of 0.05, and cells were grown at 37°C. *S. aureus* cells were harvested at mid-exponential phase (OD_600_ of 3 after growth for ~3.5 h in 100-ml cultures), at transition phase (OD_600_ of 7.5 after growth for 8 h in 40-ml cultures), and at stationary phase (OD_600_ of 6 after growth for 24 h in 50-ml cultures). *B. subtilis* cultures were harvested at mid-exponential phase (OD_600_ of 2 after growth for ~4 h in 150-ml cultures), at early stationary phase (OD_600_ of 4 after growth for 10 h in 75-ml cultures), and at stationary phase (OD_600_ of 3 after growth for 24 h in 100-ml cultures). *E. coli* cells were harvested at mid-exponential phase (OD_600_ of 1.5 after growth for ~3 h in 200-ml cultures) and at transition phase (OD_600_ of 3.5 after growth for 6.5 h in 86-ml cultures). Bacteria were centrifuged at 3,000 × *g* for 10 min and washed with 20 ml deionized water, and pellets were frozen at −80°C. Frozen cell samples were thawed at room temperature and suspended in water to yield 1.2-ml cell suspensions with an OD_600_ of 250.

Approximately 10^8^ spores of *S. coelicolor* M145 wild type and the Δ*murQ* (Δ*SCO4307*) mutant were incubated in LB medium (50 ml) and grown for 24 h in a rotary shaker at 30°C. Fifteen-milliliter amounts of the cultures were harvested by centrifugation, washed, and frozen at −80°C until being dissolved in 1,000 µl water for further analysis.

The whole bacterial samples, suspended in the amount of water indicated above, were transferred to new tubes containing ~0.25 g glass beads (0.25 to 0.5 mm; Roth), and cells were disrupted in a FastPrep FP120 (Thermo Savant) cell disrupter at speed 6 for 35 s. This was repeated 4 times, with cooling on ice for 1 min after the second cycle. Lysates were cooled briefly and subsequently centrifuged for 10 min at maximum speed in a microcentrifuge. Two hundred microliters of the supernatant was added to 800 µl of ice-cold acetone to precipitate the remaining proteins in the supernatant. After centrifugation (12,000 × *g* for 10 min), the supernatant was transferred to a new tube, and samples were dried under vacuum for 2 h at 55°C and finally stored at 4°C prior to LC-MS measurements.

### Analysis of MurNAc-6P accumulation by LC-MS.

Sample analysis of bacterial cytosolic fractions was conducted using an electrospray ionization-time of flight (ESI-TOF) mass spectrometer (MicrO-TOF II; Bruker), operated in negative-ion mode and connected to the UltiMate 3000 high-performance liquid chromatography (HPLC) system (Dionex). Dried samples were dissolved in 100 µl of water before measurement, and 5-µl amounts were injected into a Gemini C_18_ column (150 by 4.6 mm; Phenomenex) at 37°C. The following previously described (12) 45-min-gradient program at a flow rate of 0.2 ml/min was used. Five minutes of washing with 100% buffer A (0.1% formic acid with 0.05% ammonium formate) was followed by a linear gradient over 30 min to 40% buffer B (100% acetonitrile). A 5-min delay and 5 min of reequilibration completed the method. The mass spectra of the investigated samples are presented as total-ion chromatograms (TIC) and extracted-ion chromatograms (EIC) for MurNAc-6P, created using Data Analysis (Bruker) and Prism 6 (GraphPad) software. To quantify MurNAc-6P concentrations in cell extracts (presented as nmol/ml of OD1 cells), we generated EIC for MurNAc-6P (*m*/*z*^−1^ =372.07) in each sample and determined the area under the curve of the peak obtained by using Prism 6 (baseline of 30). A dilution series of a MurNAc-6P standard (1,250 µM to 2.45 µM) was also analyzed by LC-MS, and the data are presented as EIC based on the area under the curve of the measurements obtained. The standard curve was applied to define the intracellular MurNAc-6P concentrations in the bacterial cytosolic fractions.

## SUPPLEMENTAL MATERIAL

Text S1  Construction of mutants and plasmids. Download Text S1, DOCX file, 0.1 MB

Figure S1  PCR controls of recycling mutants generated in this study. Genomic DNA was isolated from *S. aureus* (*Sa*), *B. subtilis* (*Bs*), and *S. coelicolor* (*Sc*) wild-type parental strains (WT) and Δ*murQ* mutants (formerly designated *S. aureus* Δ*SAUSA*_*0193*, *B. subtilis* Δ*ybbI*, and *S. coelicolor* Δ*SCO4307*), as well as the Δ*murQPR* mutant from *S. aureus* (Δ*SAUSA*_*0192–0195*) and the Δ*murQRP* mutant from *B. subtilis*. Chromosomal regions of interest were amplified by PCR, using primers listed in Table S1 in the supplemental material, and the expected sizes in base pairs (bp) are indicated. Download Figure S1, DOCX file, 1.1 MB

Figure S2  Dilutions of MurNAc-6P standards measured by LC-MS. MurNAc-6P was generated enzymatically using MurNAc kinase according to the method in Reith et al. (31). MurNAc-6P was further purified by HPLC and quantified by a coupled enzymatic assay (S. Unsleber, M. Borisova, and C. Mayer, unpublished data). (A) Amounts of 5 µl of dilution series of the standard with concentrations from 2.5 µM to 1,250 µM were analyzed by HPLC-MS operated in negative-ion mode. Extracted-ion chromatograms (EIC) for MurNAc-6P (*m*/*z*^−1^ = 372.07) were obtained using Data Analysis software (Bruker), and the area under the curve of each sample (baseline, 30) was determined with Prism 6 software (GraphPad). (B) An example of an EIC profile (×10^3^ counts per s [cps]) of a MurNAc-6P standard with a concentration of 156 µM (*m*/*z*^−1^ = 372.07 and retention time on the HPLC column of 21 min) is presented in blue. An area under the curve of 9546 (integral of the EIC) was determined for this standard. The MurNAc-6P standard curve obtained was then used to define unknown MurNAc-6P concentrations in the cytosolic preparations of *S. aureus*, *B. subtilis*, and *E. coli* cells in different growth phases (31). Download Figure S2, DOCX file, 0.1 MB

Figure S3  Growth phase-dependent accumulation of MurNAc-6P in recycling mutants*.* Wild-type **(**WT) and Δ*murQ* cells of *S. aureus* (*Sa*) (A), *B. subtilis* (*Bs*) (B), and *E. coli* (*Ec*) (C) were grown in LB to mid-exponential (exp.) and transition (transition) growth phase. MurNAc-6P accumulation in cytosolic fractions was analyzed by LC-MS. Data for MurNAc-6P are presented with total-ion chromatograms (TIC) (×10^5^ counts per s [cps]) in gray and extracted-ion chromatogram (EIC) (×10^3^ cps) in blue (in negative-ion mode, *m*/*z*^−1^ = 372.07 and retention time of 21 min). The amounts of MurNAc-6P (nmol/ml of OD1 cells) in the Δ*murQ* strains of *B. subtilis* and *S. aureus* in exponential and transition phase, respectively, are presented as the mean values ± standard errors of the means (SEM) from four biological replicates. Download Figure S3, DOCX file, 0.4 MB

Figure S4  LC-MS analysis of MurQ-complemented *S. aureus* and *B. subtilis* Δ*murQ* mutants. Left, *S. aureus* (*Sa*) Δ*murQ* mutant transformed with empty plasmid (Δ*murQ* + pRB474) or with MurQ-expressing plasmid (ΔmurQ + pRB474-*murQ*); right, *B. subtilis* (*B*s) Δ*murQ* mutant with pX (Δ*murQ* pX) or pX-*murQ* (Δ*murQ* pX-*murQ*) construct integrated into the *amyE* site was grown for 24 h in LB medium. Cytosolic fractions were generated and analyzed by LC-MS in negative-ion mode. MS spectra for MurNAc-6P are presented with total-ion chromatograms (TIC) (×10^5^ counts per s [cps]) in gray and extracted-ion chromatograms (EIC) (×10^3^ cps) in blue (*m*/*z*^−1^ = 372.07 and retention time of 21 min). Download Figure S4, DOCX file, 0.1 MB

Figure S5  MurNAc-6P accumulation in *S. aureus* and *B. subtilis* Δ*murQ* mutants grown in LB supplemented with MurNAc. Wild-type (WT) and Δ*murQ* mutant cells of *S. aureus* (*Sa*) (A) and *B. subtilis* (*Bs*) (B) were grown in LB with 0.02% MurNAc to mid-exponential (exp.), transition (transition), and stationary (stat.) growth phases. Cytosolic fractions were generated and analyzed by LC-MS in negative-ion mode. Total-ion chromatograms (TIC) (×10^5^ counts per s [cps]) in gray and extracted-ion chromatograms (EIC) (×10^3^ cps) (*m*/*z*^−1^ = 372.07 and retention time of 21 min) in blue are shown for MurNAc-6P. MS spectra were processed in Prism 6 software (GraphPad). The amounts of MurNAc-6P detected in the Δ*murQ* strains of *S. aureus* and *B. subtilis* grown in the presence of MurNAc to exponential, transition, and stationary phase are shown as mean values (nmol/ml of OD1 cells) ± SEM from four biological replicates. Download Figure S5, DOCX file, 0.3 MB

Table S1  Primers used in this study. Table S1, DOCX file, 0.1 MB

Table S2  Strains and plasmids used in this study. Table S2, DOCX file, 0.1 MB

Table S3  Determination of the titers of viable *S. aureus* and *B. subtilis* wild-type (WT) parental and Δ*murQ* cells grown in LB medium with or without MurNAc. *S. aureus* wild-type and Δ*murQ* mutant cells were grown in LB medium in the absence or presence of 0.2% MurNAc. Viable cells were determined by counting CFU/ml (×10^8^) at mid-exponential (4 h), transition (8 h), and stationary (24 h, 48 h, and 72 h) phase. *B. subtilis* wild-type and Δ*murQ* cells were grown in LB medium in the absence or presence of 0.2% MurNAc to mid-exponential (4.5 h), transition (10 h), and stationary (24 h, 48 h, and 72 h) phase. Viable cell counts are presented as mean values ± SEM from three biological replicates. Table S3, DOCX file, 0.1 MB
