# Supplementary material for: Peptidoglycan Recycling in Gram-Positive Bacteria Is Crucial for Survival in Stationary Phase
Source: mBio. 2016 Oct 11;7(5):e00923-16. doi: 10.1128/mBio.00923-16 (PMC5061867; doi:10.1128/mBio.00923-16)
Supplement: Text S1 — Construction of mutants and plasmids. Download [file mbo005163019s1.docx]

**Supplemental Materials & Methods**

**Construction of *mutants and plasmids***

***Construction of mutants***

For the generation of a markerless *S. aureus* USA300 JE2 ∆*murQ* deletion mutant, a ~1000 bp upstream fragment (primers *SAUSA300*_*0192*_Fw and *SAUSA300*_*0192*_Rev, including the first 19 bp of the *SAUSA300_0193* gene) and a ~1000 bp downstream fragment (primers *SAUSA300*_*0194*_Fw, including the final 34 bp of the *SAUSA300_0193 gene*, and *SAUSA300*_*0194*_Rev), were amplified from genomic DNA. PCR amplicons were digested respectively with EcoRI/SacI or BglI/EcoRV, respectively, and cloned into the suicide integration vector pBASE6 that contains an erythromycin B resistance (Erm^r^) cassette flanked by *lox66* and *lox71* sites (1). pBASE6 plasmid is based on the previously described pBT2 vector but carries an additional counter selection system against the plasmid, the anhydrotetracycline-inducible expression of *secY* antisense RNA of the pKOR1 vector (2). Recombinant plasmid pBASE6-*0192*'-Erm-*0194*' was constructed and the plasmid sequence was verified by PCR and sequencing (MWG Eurofins) (data not shown). Subsequently, the plasmid was transformed into *E. coli* DC10B, which is deficient in *dcm* methylation and allows direct transformation into staphylococcal strains (3). For plasmid transformation, electrocompetent *S. aureus* cells were generated according to the following protocol (4). The protocol for replacement of the *murQ* gene was followed as previously described (2). Cre-dependent removal of the Erm resistance cassette (5), yielded the markerless *∆SAUSA300_0193* mutant. Primers *SAUSA300*_*0193*_test_Fw and *SAUSA300*_*0193*_test_Rev were used to verify the deletion of the *S. aureus* *murQ* gene by PCR. In order to generate a markerless mutant of the complete *murQ*-operon, an about 1 kb upstream flanking region (primers *SAV0190*-up-Fw and *SAV0190*-up-Rev) and an about 1.2 kb downstream flanking region (primers *SAV0190*-do-Fw and *SAV0190-*do-Rev) of the *murQ*-operon was amplified by PCR from *S. aureus* strain SA564 chromosomal DNA. The resulting amplicons were treated with restriction enzymes EcoRI/SacI and SalI/EcoRV, respectively, and introduced into pBASE6, yielding plasmid pBASE6-*murQ*-operon. The recombinant plasmid was isolated from *E. coli* DH5α, and the DNA sequence was verified by enzyme restriction and sequencing. The knock-out plasmid was subsequently introduced into *S. aureus* RN4220 via electroporation. Deletion of the entire *murQ*-operon was achieved as described previously (1, 2). The replacement of the *murQ*-operon in the correct manner was confirmed by antibiotic resistance phenotype, PCR, and DNA sequence analyses. The mutated genomic region of the *murQ* locus from strain RN4220 was transduced into JE2 by phage transduction. Briefly, an overnight culture of the donor strain was supplemented with CaCl_2_ to a final concentration of 5 mM and aliquots were infected with serial dilutions of transducing phage Φ11, 80α and/or Φ85 and distributed on tryptic soya agar plates using LB medium with 0.6% agar containing 5 mM CaCl_2_. After confluent lysis of the bacterial cells, the phage lysate was obtained and used to transfer the mutation in recipient strains. Cre-dependent removal of the Erm resistance cassette (5), yielded the markerless *∆SAUSA300_0192-0195* mutant.

Markerless in-frame *B. subtilis* deletion mutants, *∆ybbI* (*∆murQ*) and *∆ybbIHF (∆murQRP),* were generated according to a previously described protocol (6) with slight modifications. For the former mutant a ~290 bp upstream fragment (primers pJM103*ybbI*1_Fw and pJM103*ybbI*1_Rev, including the start codon of *ybbI*) and a ~350 bp downstream fragment (primers pJM103*ybbI*2_Fw, including the stop codon of *Bs* *murQ* gene, and pJM103*ybbI*2_Rev) were amplified from *B. subtilis* 168 genomic DNA. For the latter mutant a ~500 bp fragment upstream of *murQ* (primers pJMOp-BamHI-Fw and pJMOp-Hind3-Rev, without start codon of *ybbI*) and a ~500 bp fragment downstream of *ybbF* (primers pJMOp-XmaI-Fw and pJMOp-BamHI-Rev, without the intergenic region between *ybbF* and *ybbE* and without stop codon of *ybbF*) were amplified using *B. subtilis* 168 genomic DNA. Amplicons were digested with XmaI/BamHI or BamHI/HindIII, respectively, and cloned into the suicide integration vector pJM103 I-SceI, containing the endonuclease I-SceI restriction site (7), yielding vectors pJM103-I-SceI-*ybbJ'-∆ybbI*-*ybbH'* and pJM103-I-SceI-*murQ*-operon, respectively, that were isolated from Amp^r^ DH5α cells. The plasmids were transformed in chemically competent *B. subtilis* using either Spizizen (8) or MGE medium (9), respectively, and the vectors were allowed to integrate in the chromosome by a single crossover recombination event. Positive recombinant clones were selected for Cam^r^ and were verified by PCR for integration of the Cam cassette and for integration of the suicide plasmid (data not shown). To remove the pJM103 plasmid intergrates from the genome, a second plasmid (pBKJ223), expressing the endonuclease I-SceI (6) was co-transformed and selected for Tc^r^. Expressed I-SceI resulted in a double-strand break in the DNA, which is repaired by homologous recombination either yielding a markerless deletion or regaining wild-type cells. Single colonies were screened for Tc^r^ and Cam^s^ and controlled by PCR and genomic sequencing for *murQ* gene deletion (primers A_Fw_Cluster1 and C_Rev_Cluster1) and *murQRP* operon deletion (primers pJMOp-XmaI-Fw and pJMOp-HindIII-Rev). Finally, *B. subtilis* ∆*murQ* and Δ*murQRP* mutants were cured for the pBKJ223 plasmid by serial transfers on LB plates, followed by screening for Tc^s^ phenotype.

To inactivate gene *SCO4307*, a ~1.5 kb upstream fragment (primer pair up*4307*FwE and up*4307*RevH), including the start codon of *SCO4306* and a ~1.5 kb downstream fragment (primer pair nlo*4307*FwH/nlo*4307*RevB), including the 3´end of *SCO4307*, were amplified from *S. coelicolor* M145 genomic DNA, digested with EcoRI/HindIII and HindIII/BamHI, respectively, and cloned into pGus21 plasmid (G. Muth, unpublished). The resulting deletion plasmid pKO4307 was introduced into *S. coelicolor* M145 by intergeneric conjugation and apramycin resistant transconjugants were selected that carried pKO4307 integrated via a single crossover (M145::pKO4307). To screen for the second cross over, resulting in the *S. coelicolor ∆SCO4307* deletion mutant*,* M145::pKO4307 was plated onto soja mannit (SM) agar without antibiotic and incubated for five days. Spores were harvested and appropriate dilutions were plated onto LB agar to obtain single colonies. After two days incubation at 30°C, plates were overlaid with 1 ml H_2_O containing 2.5 mg X-glucuronide (5-bromo-4-chloro-3-indolyl glucuronide). Colonies that still carried pKO4307 were surrounded by a blue halo due to 4-Cl-3-Br-indigo production by the α-glucuronidase (GusA). Colonies that had lost pKO4307 by a second crossover event were identified by the lack of the blue halo. Deletion of *S. coelicolor* *murQ gene* was confirmed by PCR analysis using primers Kn*4307*Fw and Kn*4307*Rev.

**MurQ complementation**

*S. aureus* JE2 ∆*murQ* mutant was complemented *in trans* using *the E. coli* - *S. aureus* shuttle vector *pRB474,* which constitutively expresses the *SAUSA300*_*0193* (*murQ*) gene from the veg II promoter (10). *S. aureus* *murQ* gene, including 50 bp of the 3’ end of the upstream gene *sausa300*_*0192* was PCR-amplified from the isolated JE2 genomic DNA (primers pRB474_*0193*_Fw and pRB474_*0193*_Rev), introducing a HindIII restriction site at the 5’ end and an EcoRI restriction site at the 3’ end of the primers. The amplified fragment was subcloned into HindIII/EcoRI-digested pRB474. The resulting plasmid pRB474-*murQ* was isolated from Amp^r^ DC10B cells and verified by EcoRI/BamHI enzyme restriction and sequencing (primers pRB474_*0193*_test_Fw and pRB474_*0193*_test_Rev). Plasmid pRB474-*murQ* was transformed in electrocompetent *S. aureus* wild-type and ∆*murQ* cells, and correct clones were confirmed by antibiotic phenotype, restriction enzyme analyses, and sequencing (data not shown).

Plasmid pX with a xylose-inducible promoter was used to complement the *B. subtilis* ∆*murQ* mutant with the *ybbI* (*murQ*) gene. *YbbI* gene was amplified by PCR using primers pX_*murQ*_Fw and pX_*murQ*_Rev with BamHI restriction sites. Amplified fragments were inserted in the BamHI restriction site of pX plasmid, producing pX-*murQ* construct, which was subsequently transformed in chemically competent (by the method of Spizizen) *B. subtilis* cells. The *B. subtilis* *murQ* gene integrated at the *amyE* locus by a double cross-over event, which was confirmed by blue staining with iodine solution and PCR with primers, specific for the *amyE* site and cloning primers (data not shown).

**References**

1. **Geiger T, Francois P, Liebeke M, Fraunholz M, Goerke C, Krismer B, Schrenzel J, Lalk M, Wolz C.** 2012. The stringent response of *Staphylococcus aureus* and its impact on survival after phagocytosis through the induction of intracellular PSMs expression. PLoS Pathog **8:**e1003016.

2. **Bae T, Schneewind O.** 2006. Allelic replacement in *Staphylococcus aureus* with inducible counter-selection. Plasmid **55:**58-63.

3. **Monk IR, Shah IM, Xu M, Tan MW, Foster TJ.** 2012. Transforming the untransformable: application of direct transformation to manipulate genetically *Staphylococcus aureus* and *Staphylococcus epidermidis*. MBio **3**.

4. **Löfblom J, Kronqvist N, Uhlén M, Ståhl S, Wernérus H.** 2007. Optimization of electroporation-mediated transformation: *Staphylococcus carnosus* as model organism. J Appl Microbiol **102:**736-747.

5. **Leibig M, Krismer B, Kolb M, Friede A, Götz F, Bertram R.** 2008. Marker removal in staphylococci via Cre recombinase and different lox sites. Appl Environ Microbiol **74:**1316-1323.

6. **Janes BK, Stibitz S.** 2006. Routine markerless gene replacement in *Bacillus anthracis*. Infect Immun **74:**1949-1953.

7. **Szurmant H, Mohan MA, Imus PM, Hoch JA.** 2007. YycH and YycI interact to regulate the essential YycFG two-component system in *Bacillus subtilis*. J Bacteriol **189:**3280-3289.

8. **Spizizen J, Reilly BE, Evans AH.** 1966. Microbial transformation and transfection. Annu Rev Microbiol **20:**371-400.

9. **Msadek T, Kunst F, Henner D, Klier A, Rapoport G, Dedonder R.** 1990. Signal transduction pathway controlling synthesis of a class of degradative enzymes in *Bacillus subtilis*: expression of the regulatory genes and analysis of mutations in *degS* and *degU*. J Bacteriol **172:**824-834.

10. **Brückner R.** 1992. A series of shuttle vectors for *Bacillus subtilis* and *Escherichia coli*. Gene **122:**187-192.
