# Supplementary material for: Peptidoglycan Recycling in Gram-Positive Bacteria Is Crucial for Survival in Stationary Phase
Source: mBio. 2016 Oct 11;7(5):e00923-16. doi: 10.1128/mBio.00923-16 (PMC5061867; doi:10.1128/mBio.00923-16)
Supplement: Figure S1 — PCR controls of recycling mutants generated in this study. Genomic DNA was isolated from S. aureus (Sa), B. subtilis (Bs), and S. coelicolor (Sc) wild-type parental strains (WT) and ΔmurQ mutants (formerly designated S. aureus ΔSAUSA_0193, B. subtilis ΔybbI, and S. coelicolor ΔSCO4307), as well as the ΔmurQPR mutant from S. aureus (ΔSAUSA_0192–0195) and the ΔmurQRP mutant from B. subtilis. Chromosomal regions of interest were amplified by PCR, using primers listed in Table S1 in the supplemental material, and the expected sizes in base pairs (bp) are indicated. Download [file mbo005163019sf1.docx]

**Figure S1 PCR controls of recycling mutants generated in this study.** Genomic DNA was isolated from *S. aureus* (*Sa*), *B. subtilis* (*Bs*) and *S. coelicolor* (*Sc*) wild-type parental strains (WT) and ∆*murQ* mutants (formerly designated *Sa* ∆*SAUSA_0193, Bs* ∆*ybbI* and *Sc* ∆*SCO4307*), as well as the ∆*murQPR* mutant from *S. aureus* (∆*SAUSA*_*0192-0195)* and the ∆*murQRP* mutant from *B. subtilis*. Chromosomal regions of interest were amplified by PCR, using primers listed in Table S1 in the supplemental material, and the expected sizes in base pairs (bp) are indicated.
