# Supplementary material for: Peptidoglycan Recycling in Gram-Positive Bacteria Is Crucial for Survival in Stationary Phase
Source: mBio. 2016 Oct 11;7(5):e00923-16. doi: 10.1128/mBio.00923-16 (PMC5061867; doi:10.1128/mBio.00923-16)
Supplement: Figure S2 — Dilutions of MurNAc-6P standards measured by LC-MS. MurNAc-6P was generated enzymatically using MurNAc kinase according to the method in Reith et al. (31). MurNAc-6P was further purified by HPLC and quantified by a coupled enzymatic assay (S. Unsleber, M. Borisova, and C. Mayer, unpublished data). (A) Amounts of 5 µl of dilution series of the standard with concentrations from 2.5 µM to 1,250 µM were analyzed by HPLC-MS operated in negative-ion mode. Extracted-ion chromatograms (EIC) for MurNAc-6P (m/z−1 = 372.07) were obtained using Data Analysis software (Bruker), and the area under the curve of each sample (baseline, 30) was determined with Prism 6 software (GraphPad). (B) An example of an EIC profile (×103 counts per s [cps]) of a MurNAc-6P standard with a concentration of 156 µM (m/z−1 = 372.07 and retention time on the HPLC column of 21 min) is presented in blue. An area under the curve of 9546 (integral of the EIC) was determined for this standard. The MurNAc-6P standard curve obtained was then used to define unknown MurNAc-6P concentrations in the cytosolic preparations of S. aureus, B. subtilis, and E. coli cells in different growth phases (31). Download [file mbo005163019sf2.docx]

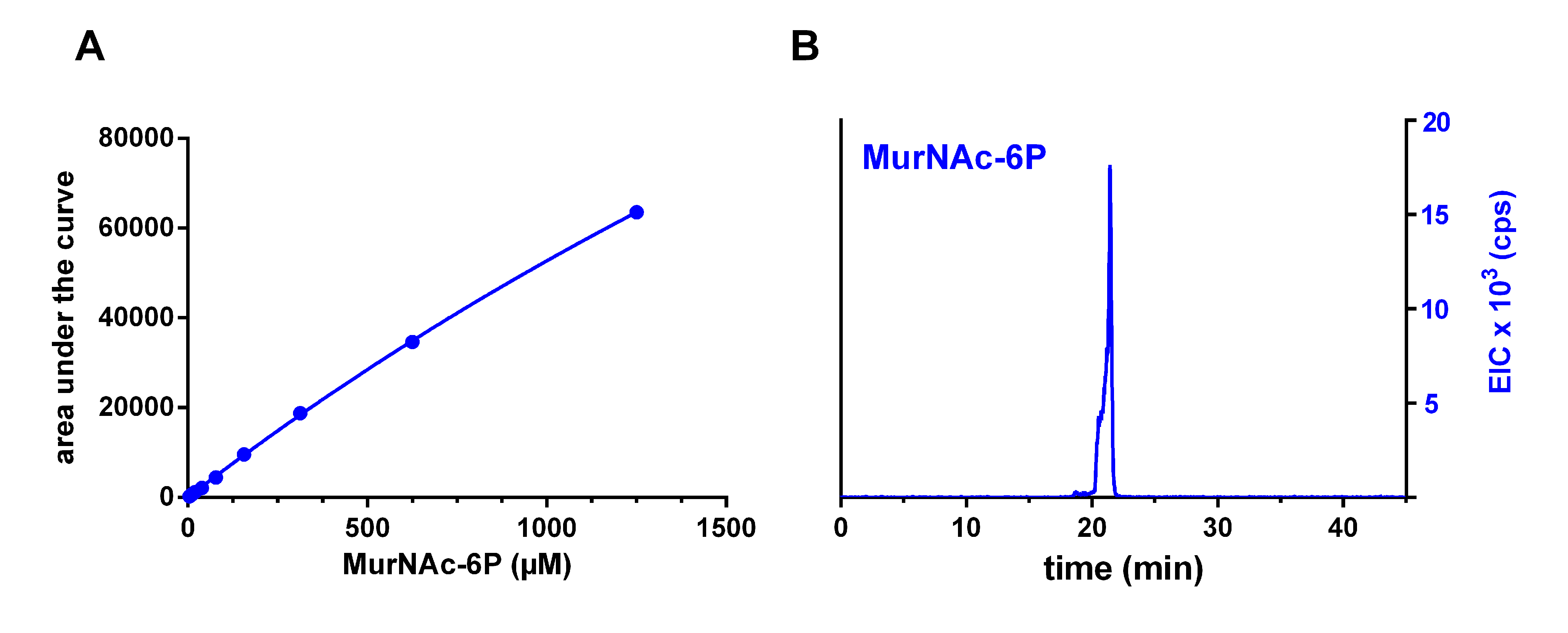
**Figure S2. Dilutions of MurNAc-6P standards measured by LC-MS.** MurNAc-6P was generated enzymatically using MurNAc kinase according to (1). MurNAc-6P was further purified by HPLC and quantified by a coupled enzymatic assay (Unsleber and Mayer, manuscript in preparation). A, 5 µl of dilution series of the standard with concentrations from 2.5 µM to 1250 µM were analyzed by HPLC-MS operated in negative ion mode. Extracted ion chromatograms (EIC) for MurNAc-6P (m/z^-1^ =372.07) were obtained using the program Data Analysis (Bruker) and the area under the curve of each sample (baseline 30) was determined with the program Prism 6 (GraphPad). B, Example of an EIC profile x 10^3^ counts per second (cps) of MurNAc-6P standard with concentration of 156 µM (m/z^-1^ =372.07 and retention time on the HPLC column of 21 min) is presented in blue. Area under curve of 9546 was determined for this standard. Obtained MurNAc-6P standard curve was then used to define unknown MurNAc-6P concentrations in the cytosolic preparations of *S. aureus* and *B. subtilis* and *E. coli* cells in different growth phases.

**Reference:
(**1) Reith J, Berking A, Mayer C. 2011. Characterization of an N-acetylmuramic acid/N-acetyl-glucosamine kinase of *Clostridium acetobutylicum*. Journal of bacteriology 193:5386-5392.
