# Supplementary material for: Peptidoglycan Recycling in Gram-Positive Bacteria Is Crucial for Survival in Stationary Phase
Source: mBio. 2016 Oct 11;7(5):e00923-16. doi: 10.1128/mBio.00923-16 (PMC5061867; doi:10.1128/mBio.00923-16)
Supplement: Figure S3 — Growth phase-dependent accumulation of MurNAc-6P in recycling mutants. Wild-type (WT) and ΔmurQ cells of S. aureus (Sa) (A), B. subtilis (Bs) (B), and E. coli (Ec) (C) were grown in LB to mid-exponential (exp.) and transition (transition) growth phase. MurNAc-6P accumulation in cytosolic fractions was analyzed by LC-MS. Data for MurNAc-6P are presented with total-ion chromatograms (TIC) (×105 counts per s [cps]) in gray and extracted-ion chromatogram (EIC) (×103 cps) in blue (in negative-ion mode, m/z−1 = 372.07 and retention time of 21 min). The amounts of MurNAc-6P (nmol/ml of OD1 cells) in the ΔmurQ strains of B. subtilis and S. aureus in exponential and transition phase, respectively, are presented as the mean values ± standard errors of the means (SEM) from four biological replicates. Download [file mbo005163019sf3.docx]

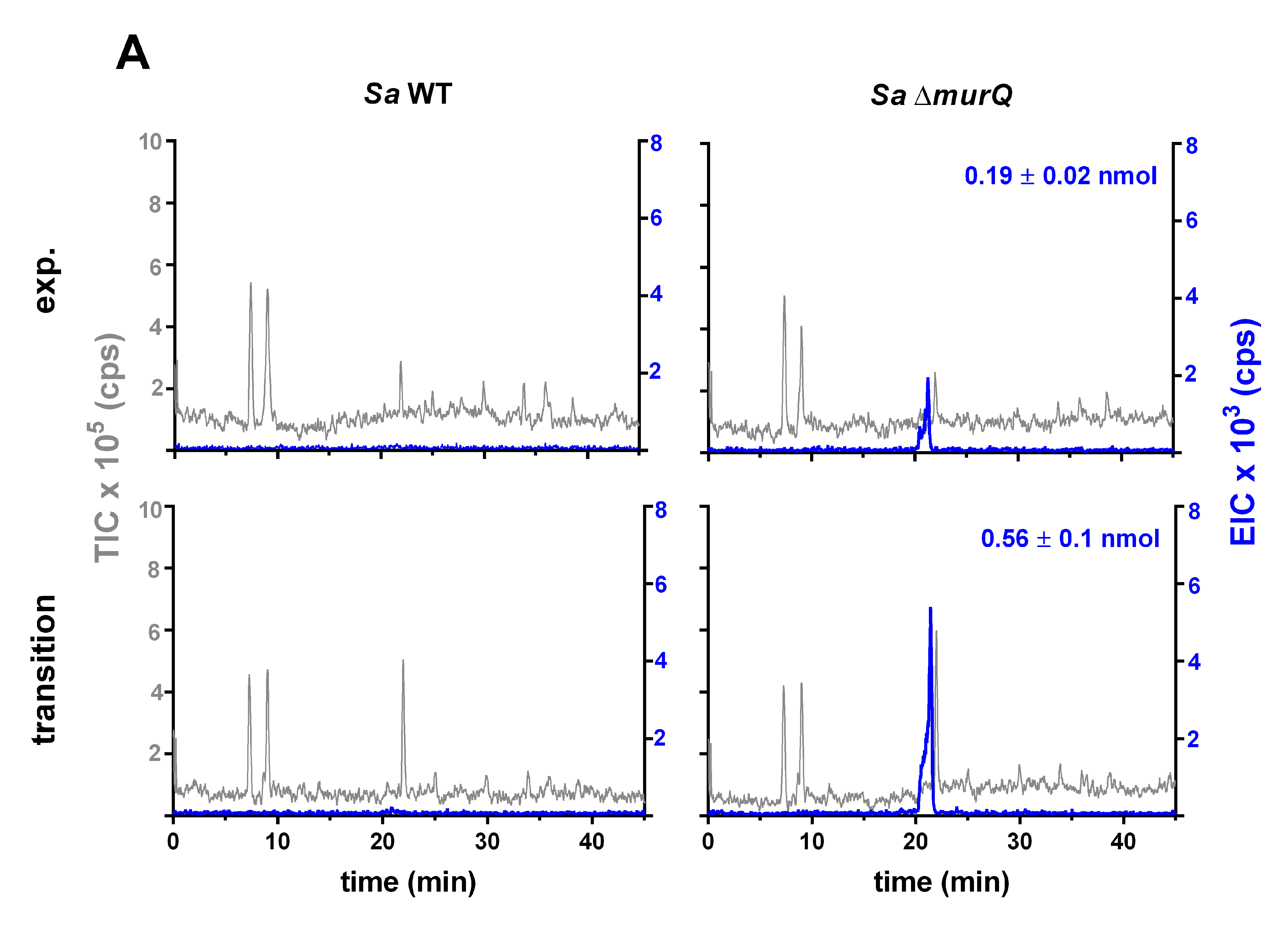

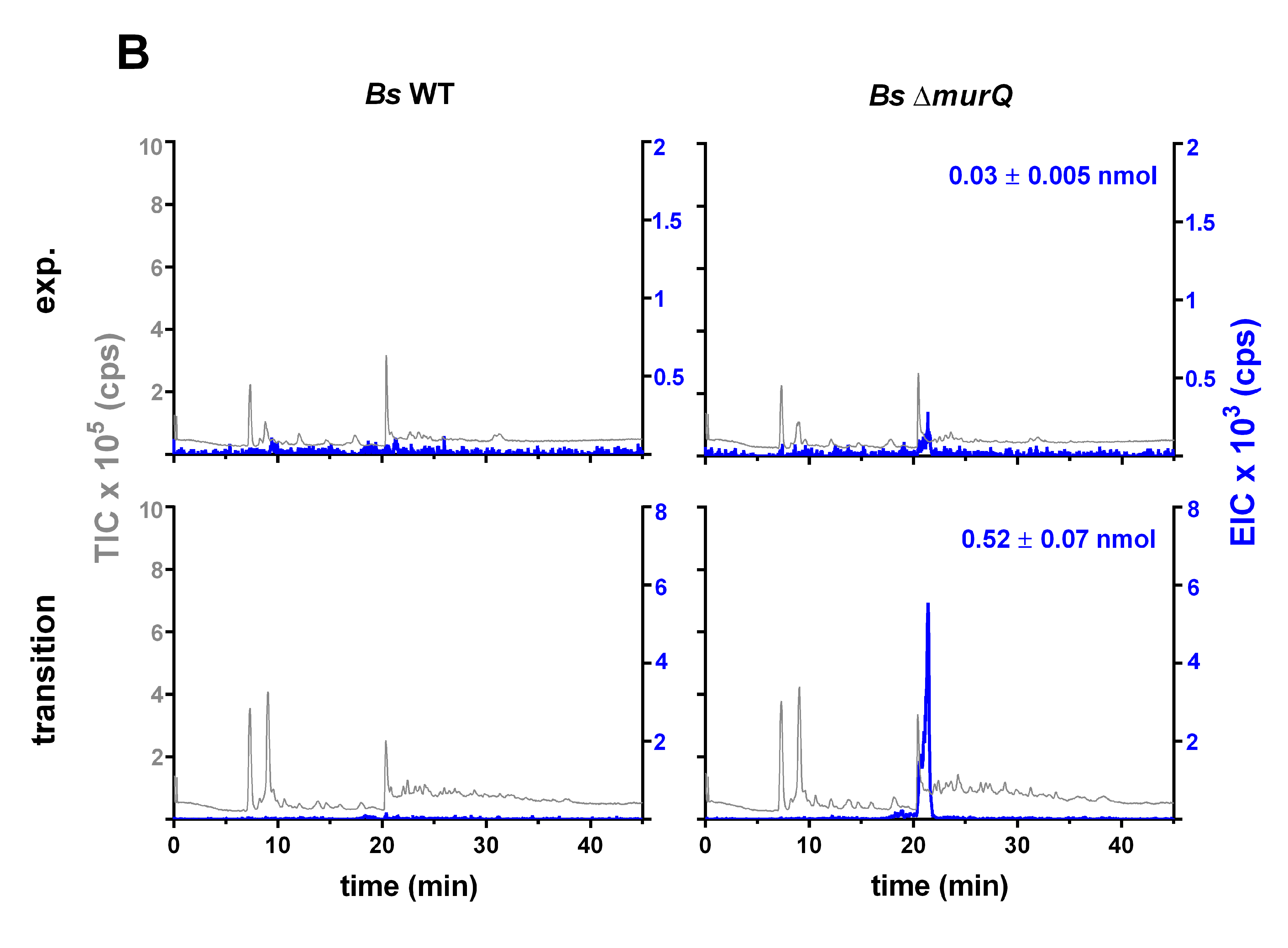

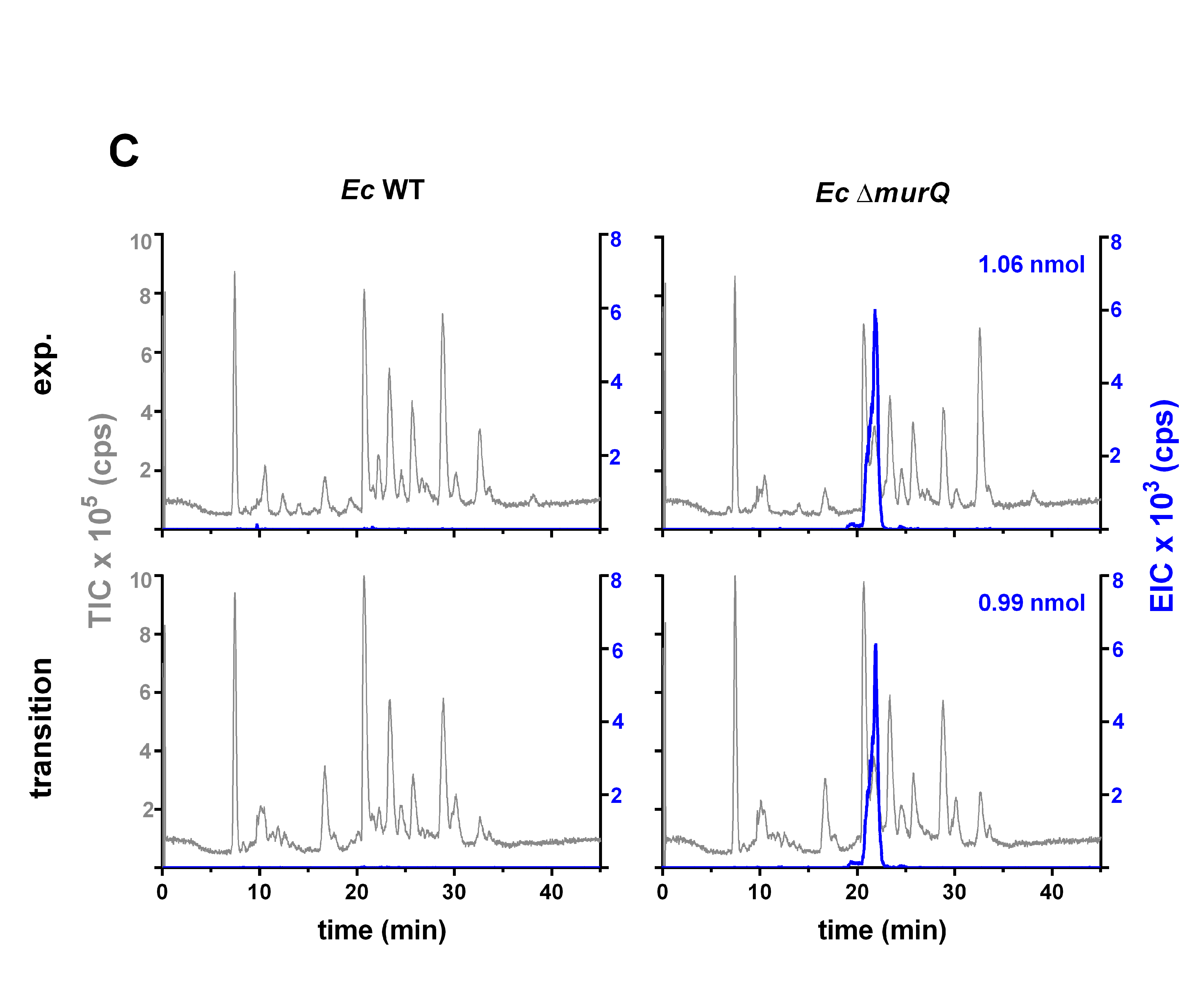


**Figure S3. Growth phase-dependent accumulation of MurNAc-6P in recycling mutants*.*** Wild-type **(**WT) and ∆*murQ* cells of *A*, *S. aureus* (*Sa*), *B*, *B. subtilis* (*Bs*), and *C*, *E. coli* (*Ec*), were grown in LB to mid exponential (exp.) and transition growth phase (transition). MurNAc-6P accumulation in cytosolic fractions was analyzed by LC-MS. Data were presented as total ion chromatograms (TIC) x 10^5^ counts per second (cps) in grey and extracted ion chromatogram (EIC) x 10^3^ cps in blue, for MurNAc-6P (m/z^-1^ = 372.07, in negative ion mode, retention time of 21 min). The determined amounts of MurNAc-6P in nmol/OD 1 cells in the ∆*murQ* strains of *Bs* and *Sa* in exp. and transition phase, respectively, were indicated as mean ± SEM from four biological replicates.
