# Supplementary material for: Peptidoglycan Recycling in Gram-Positive Bacteria Is Crucial for Survival in Stationary Phase
Source: mBio. 2016 Oct 11;7(5):e00923-16. doi: 10.1128/mBio.00923-16 (PMC5061867; doi:10.1128/mBio.00923-16)
Supplement: Figure S4 — LC-MS analysis of MurQ-complemented S. aureus and B. subtilis ΔmurQ mutants. Left, S. aureus (Sa) ΔmurQ mutant transformed with empty plasmid (ΔmurQ + pRB474) or with MurQ-expressing plasmid (ΔmurQ + pRB474-murQ); right, B. subtilis (Bs) ΔmurQ mutant with pX (ΔmurQ pX) or pX-murQ (ΔmurQ pX-murQ) construct integrated into the amyE site was grown for 24 h in LB medium. Cytosolic fractions were generated and analyzed by LC-MS in negative-ion mode. MS spectra for MurNAc-6P are presented with total-ion chromatograms (TIC) (×105 counts per s [cps]) in gray and extracted-ion chromatograms (EIC) (×103 cps) in blue (m/z−1 = 372.07 and retention time of 21 min). Download [file mbo005163019sf4.docx]

**
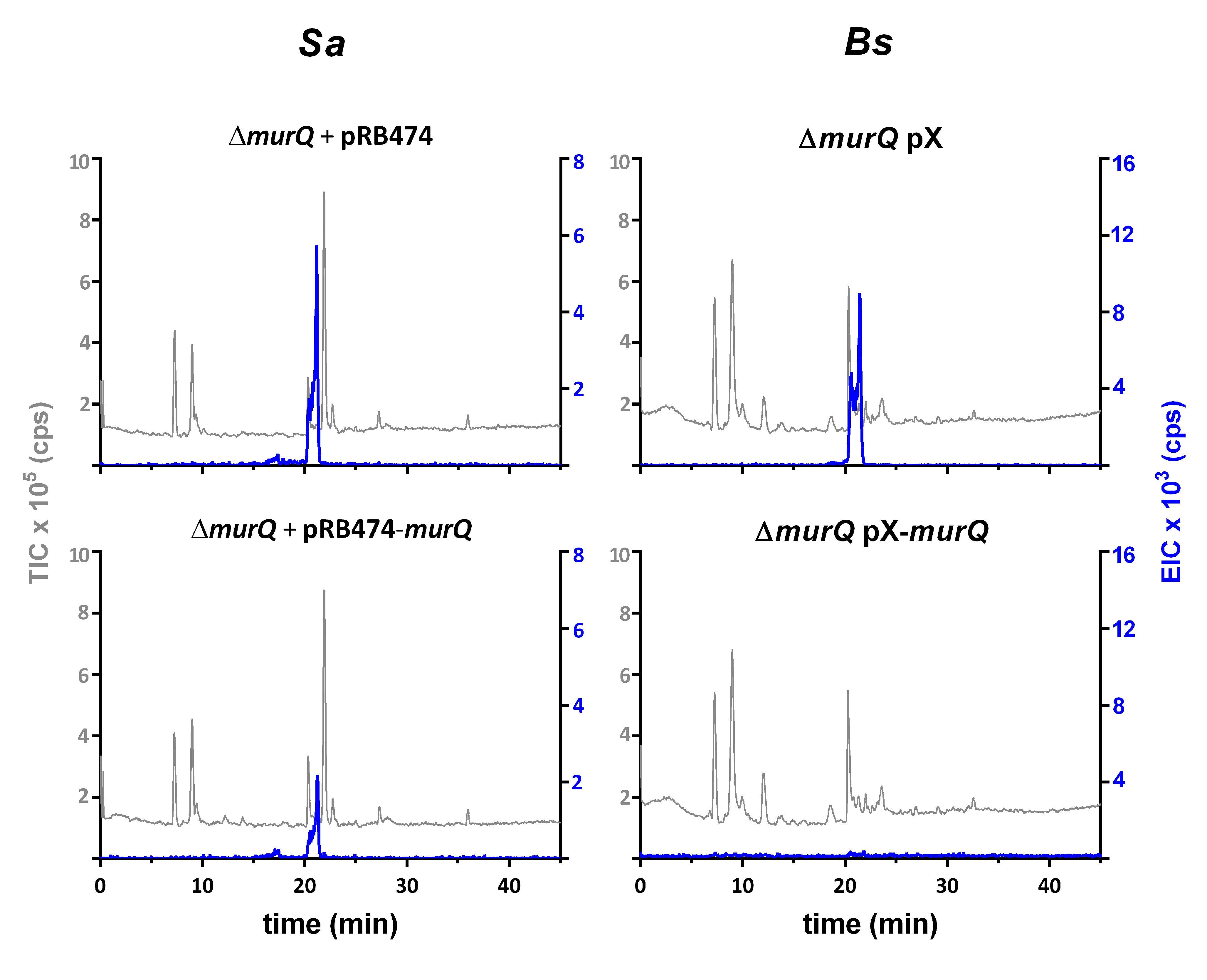
Figure S4. LC-MS analysis of MurQ complemented *S. aureus* and *B. subtilis* ∆*murQ* mutants.** Left, *S. aureus* (*Sa*) ∆*murQ* mutant, transformed with empty plasmid (∆*murQ* + pRB474) or with MurQ-expressing plasmid (∆*murQ* + pRB474-*murQ*), and right, *B. subtilis* (*Bs*) ∆*murQ* mutant with integrated in the *amyE* site pX (∆*murQ* pX) or pX-*murQ* (∆*murQ* pX-*murQ*) constructs were grown for 24 h in LB medium. Cytosolic fractions were generated and analyzed by LC-MS in negative ion mode. MS spectra are presented as total ion chromatograms (TIC) x 10^5^ cps in grey and extracted ion chromatogram (EIC) x 10^3^ cps for MurNAc-6P (m/z^-1^ =372.07, retention time of 21 min) in blue.
