# Supplementary material for: Peptidoglycan Recycling in Gram-Positive Bacteria Is Crucial for Survival in Stationary Phase
Source: mBio. 2016 Oct 11;7(5):e00923-16. doi: 10.1128/mBio.00923-16 (PMC5061867; doi:10.1128/mBio.00923-16)
Supplement: Figure S5 — MurNAc-6P accumulation in S. aureus and B. subtilis ΔmurQ mutants grown in LB supplemented with MurNAc. Wild-type (WT) and ΔmurQ mutant cells of S. aureus (Sa) (A) and B. subtilis (Bs) (B) were grown in LB with 0.02% MurNAc to mid-exponential (exp.), transition (transition), and stationary (stat.) growth phases. Cytosolic fractions were generated and analyzed by LC-MS in negative-ion mode. Total-ion chromatograms (TIC) (×105 counts per s [cps]) in gray and extracted-ion chromatograms (EIC) (×103 cps) (m/z−1 = 372.07 and retention time of 21 min) in blue are shown for MurNAc-6P. MS spectra were processed in Prism 6 software (GraphPad). The amounts of MurNAc-6P detected in the ΔmurQ strains of S. aureus and B. subtilis grown in the presence of MurNAc to exponential, transition, and stationary phase are shown as mean values (nmol/ml of OD1 cells) ± SEM from four biological replicates. Download [file mbo005163019sf5.docx]

**
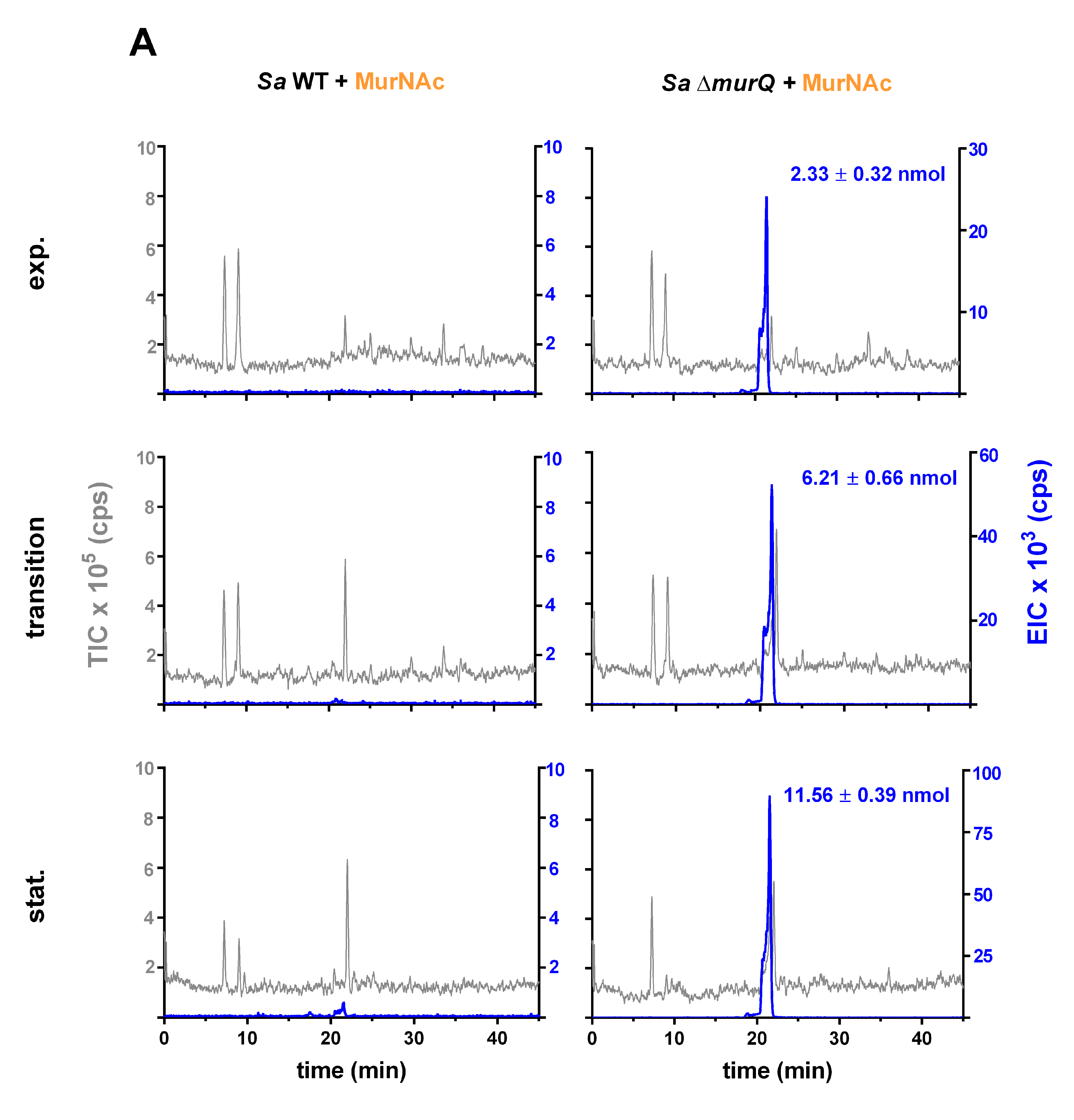
**

**
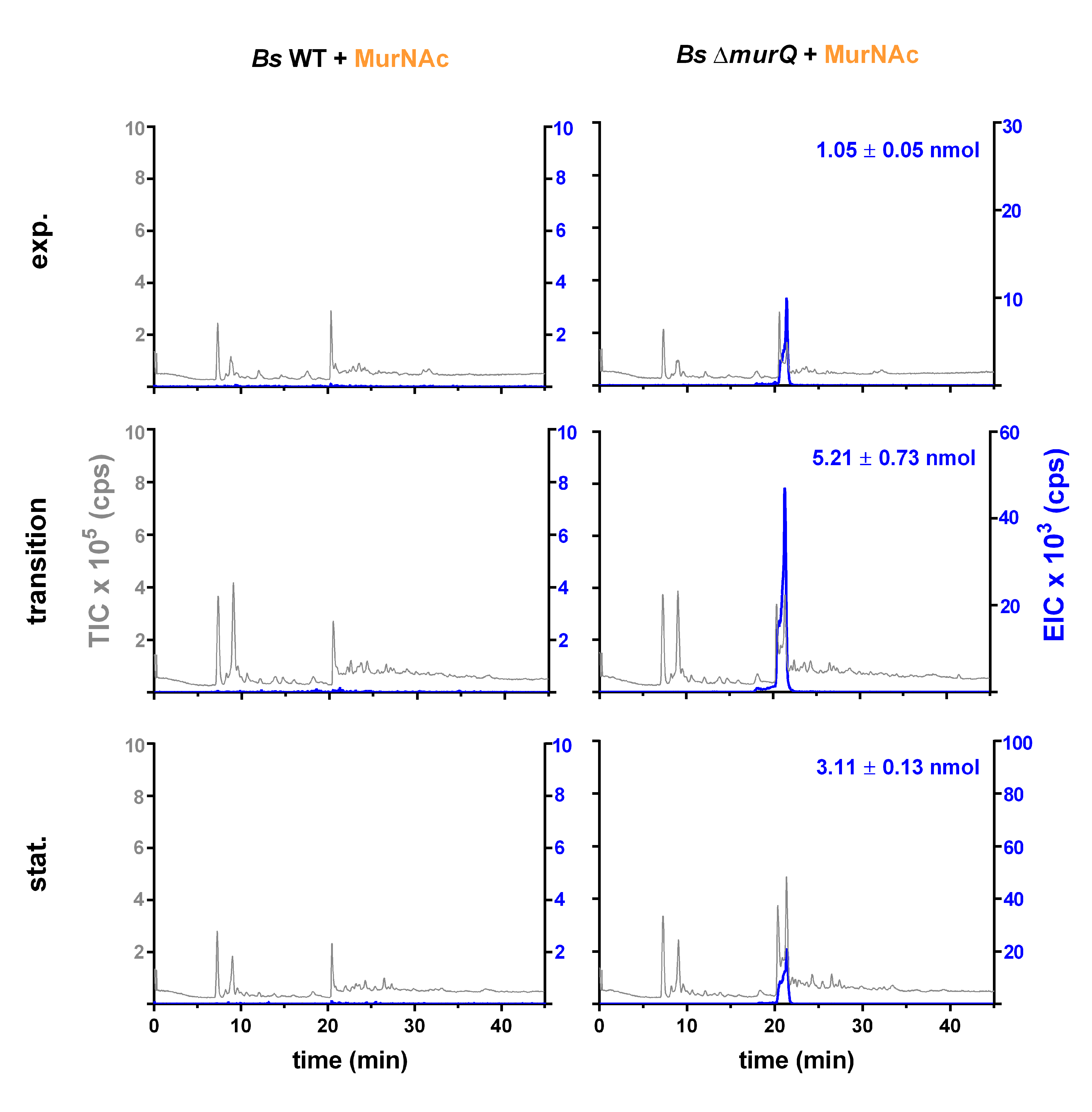

Figure S5. MurNAc-6P accumulation in *S. aureus* and *B. subtilis* ∆***murQ* **mutants, grown in LB supplemented with MurNAc.** Wild-type (WT) and ∆*murQ* mutant cells of *S. aureus* (*Sa*), *A*, and *B. subtilis (Bs), B,* were cultured in LB with 0.02% MurNAc to mid exponential (exp.), transition and stationary (stat.) growth phases. Cytosolic fractions were generated and analyzed by LC-MS in negative ion mode. Total ion chromatograms (TIC) x 10^5^ counts per second (cps) in grey and extracted ion chromatogram (EIC) x 10^3^ cps for MurNAc-6P (m/z^-1^ =372.07, retention time of 21 min) in blue. MS spectra were presented in Prism 6 (GraphPad) program. The amounts of MurNAc-6P, detected in the ∆*murQ* strains of *S. aureus* and *B. subtilis* grown in the presence of MurNAc to exp., transition, and stat. phase, respectively, were shown as mean of nmol/OD1 cells ± SEM from four biological replicates.

**B**
