# Supplementary material for: Peptidoglycan Recycling in Gram-Positive Bacteria Is Crucial for Survival in Stationary Phase
Source: mBio. 2016 Oct 11;7(5):e00923-16. doi: 10.1128/mBio.00923-16 (PMC5061867; doi:10.1128/mBio.00923-16)
Supplement: Table S1 — Primers used in this study. [file mbo005163019st1.docx]

**Table S1. Primers used in this study**

| **Primers** | **Sequence 5'-3'** | **Application** |
| --- | --- | --- |
| *SAUSA300*_*0192*_Fw | CCGGAATTCGGCTTTTCAGTGTATTTAGG | deletion of *SAUSA300*_*0193* (*murQ*) gene in *S. aureus* |
| *SAUSA300*_*0192*_Rev | GCCGAGCTCGGTCGTACTATTTTCCATCAC | deletion of *SAUSA300*_*0193* (*murQ*) gene in *S. aureus* |
| *SAUSA300*_*0194*_Fw | CCGAGATCTGTTAAACGAGCAATCAGAG | deletion of *SAUSA300*_*0193* (*murQ*) gene in *S. aureus* |
| *SAUSA300*_*0194*_Rev | GCCGATATCCTAATGCGGCACCTACTTG | deletion of *SAUSA300*_*0193* (*murQ*) gene in *S. aureus* |
| *SAUSA300*_*0193*_test_Fw | GTTATTGTAATAATCCAGCAGTAG | verification of *SAUSA300*_*0193* (*murQ*) gene deletion in *S. aureus* |
| *SAUSA300*_*0193*_test_Rev | CACCCAACAAATCTGTCTGTCG | verification of *SAUSA300*_*0193* gene (*murQ*) deletion in *S. aureus* |
| pRB474_*0193*_Fw | GAGAAGCTTGAGCCGAATGATACATTTG | cloning of *SAUSA300_0193* (*murQ*) in pRB474 in *S. aureus* |
| pRB474_*0193*_Rev | GGCGAATTCCCTCCTAAGGTTGTCTATCTC | cloning of *SAUSA300_0193* (*murQ*) in pRB474 in *S. aureus* |
| pRB474_*0193*_test_Fw | GTCTTTCGACTGAGCCTTTC | sequencing of pRB474-*murQ* in *S. aureus* |
| pRB474_*0193*_test_Rev | GCTAGAGCGGCGGATTTGTC | sequencing of pRB474-*murQ* in *S. aureus* |
| *SAV0190*-up-Fw | ATAATGAATTCATAATAGGTACGAAACGCTTAC | deletion of *SAUSA300_0192* to *SAUSA300_0195* genes |
| *SAV0190*-up-Rev | AATTAGAGCTCTAATCAGTTAAAGCCTGTTCATTG | deletion of *SAUSA300_0192* to *SAUSA300_0195* genes |
| *SAV0190*-do-Fw | AATATGTCGACCTTGATAATTACCGGAAGCATC | deletion of *SAUSA300_0192* to *SAUSA300_0195* genes |
| *SAV0190*-do-Rev | TATAAGATATCGTGTAATTTTGTTTGCGGTAACG | deletion of *SAUSA300_0192* to *SAUSA300_0195* genes |
| pJM103*ybbI*1_Fw | GCCCGGGTGCTTCCCGCTTTTGAGC | deletion of *ybbI (murQ)* gene in *B. subtilis* |
| pJM103*ybbI*1_Rev | GCGGGATCCCATGAGGATGCCCCCTGTT | deletion of *ybbI* (*murQ*) gene in *B. subtilis* |
| pJM103*ybbI*2_Fw | GCGGGATCCTGATAAGGAGAGAACTAG | deletion of *ybbI* (*murQ*) gene in *B. subtilis* |
| pJM103*ybbI*2_Rev | GAAGCTTTCCTGAATGGCTTGAATGG | deletion of *ybbI* (*murQ*) gene in *B. subtilis* |
| A_Fw_Cluster1 | GCCTGAACACTTGCCCGAGCTCCAATCA | verification of *ybbI* (*murQ*) gene deletion in *B. subtilis* |
| C_Rev_Cluster1 | CGTCACTTGAATTGGCTAAGGCGC | verification of *ybbI* (*murQ*) gene deletion in *B. subtilis* |
| pJMOp-XmaI-Fw | AGCTGCCCGGGTTCCGGCGTGTAAAAGTAAA | deletion of *murQRP* operon in *B. subtilis* |
| pJMOp-BamHI-Rev | CAGCTGGATCCGTGAAAACAAAGACACTGTT | deletion of *murQRP* operon in *B. subtilis* |
| pJMOp-BamHI-Fw | AGCTTGGATCCGAGGATGCCCCCTGTTTTAA | deletion of *murQRP* operon in *B. subtilis* |
| pJMOp-HindIII-Rev | CAGCTAAGCTTTCCAATCATTCACGCTCACC | deletion of *murQRP* operon in *B. subtilis* |
| pX-*murQ*_Fw | CGCGGATCCATGTCAGAACCATTA | Integration of *ybbI* gene in in the *amiE* site of 168 ∆*ybbI* mutant |
| pX-*murQ*_Rev | CGCGGATCCTCATGGATGGTAATGTTC | Integration of *ybbI* gene in in the *amiE* site of 168 ∆*ybbI* mutant |
| Up*4307*FwE | CCGAATTCGACGTCGACCTCCTC | deletion of *SCO4307* (*murQ*) gene in *S. coelicolor* |
| Up*4307*RevH | GCAAGCTTCATGTCACTCCCCACC | deletion of *SCO4307* (*murQ*) gene in *S. coelicolor* |
| Nlo*4307*FwH | CCAAGCTTATCCTGGTCCTGCTGG | deletion of *SCO4307* (*murQ*) gene in *S. coelicolor* |
| Nlo*4307*RevB | AAGGATCCGAACTCAAGGGCCGTG | deletion of *SCO4307* (*murQ*) gene in *S. coelicolor* |
| Kn*4307*Fw | TCACCCAGAAGATGCT | verification of *SCO4307* (*murQ*) gene deletion in *S. coelicolor* |
| Kn*4307*Rev | CTTCAGAATCGCACAC | verification of *SCO4307* (*murQ*) gene deletion in *S. coelicolor* |

Enzyme restriction sites are underlined
