# Supplementary material for: Peptidoglycan Recycling in Gram-Positive Bacteria Is Crucial for Survival in Stationary Phase
Source: mBio. 2016 Oct 11;7(5):e00923-16. doi: 10.1128/mBio.00923-16 (PMC5061867; doi:10.1128/mBio.00923-16)
Supplement: Table S2 — Strains and plasmids used in this study. [file mbo005163019st2.docx]

**Table S2. Strains and plasmids used in this study**

| **Strains and plasmids** | **Relevant properties*** | **Reference/source** | |  |
| --- | --- | --- | --- | --- |
| ***Plasmids*** |  |  |  |  |
| pBASE6 | *E. coli* – *S. aureus* temperature-sensitive suicide shuttle vector, Amp^r^,Erm^r^ | (1) | | |
| pBASE6-*0192*'-Erm-*0194*' | pBASE6 suicide integration vector for deletion of *S aureus* *SAUSA300_0193* | this study | | |
| pBASE6-*murQ-*operon | pBASE6 suicide integration vector for deletion of *S. aureus* *SAUSA300*_*0192*_*0195* | this study | | |
| pRAB1 | PpagA-cre; pBT2 derivative; expression of *cre* in staphylococci, Amp^r^,Cam^r^ | (2) | | |
| pRB474 | *E. coli* – *S. aureus* shuttle vector, transcription from veg II promoter, Amp^r^,Cam^r^ | (3) | | |
| pRB474-*murQ* | Cloning of *S. aureus* *SAUSA300_0193* (*murQ*) gene in pRB474, Amp^r^,Cam^r^ | this study | | |
| pJM103 I-SceI | Suicide integration vector with I-SceI restriction site, Amp^r^*,*Cam^r^ | (4) | | |
| pJM103 I-SceI-*ybbJ'*-∆*ybbI*-*ybbH'* | Suicide integration vector for depletion of *B.subtilis* 168 *ybbI* (*murQ*) , Amp^r^,Cam^r^ | this study | | |
| pJM103-I-SceI-*murQ*-operon | Suicide integration vector for depletion of *B.subtilis* 168 *ybbIHF* (*murQRP*) , Amp^r^,Cam^r^ | this study | | |
| pBKJ223 | I-SceI expression vector, Amp^r^,Tc^r^ | (5) | | |
| pX | Integration of genes into the *amyE* locus of *B. subtilis*; P*xylA*′ promoter, Amp^r^,Cam^r^ | (6) | | |
| pX-*murQ* | pX derivative carrying the *ybbI* (*murQ*) gene from *B. subtilis* 168 | this study | | |
| pGus21 | *E. coli* vector, *aac* (Apr^r^), *gusA,* | G. Muth, unpublished | | |
| pKO4307 | *SCO4307* (*murQ*) deletion plasmid, pGus21 derivative, *aac* (Apr^r^*), gusA,* | this study | | |
| ***Strains*** |  |  | | |
| *E. coli* DH5α | *supE44 hsdR17 recA1 endA1 gyrA96 thi-1 relA1* | (7) | | |
| *E. coli* DC10B | *E. coli* DH10B Δ*dcm* | (8) | | |
| *E. coli* DC10B + pRB474 | DC10B cells, transformed with pRB474, Amp^r^ | F. Götz laboratory | | |
| *E coli* DC10B + pRB474-*murQ* | DC10B cells, transformed with pRB474-*murQ,* Amp^r^ | this study | | |
| *E. coli* MC4100 | *F^−^araD139 Δ(argF-lac)U169 flbB5301 deoC1 relA1 rbsR rpsL150 ptsF25* | (9) | | |
| *E. coli* TJ2e | MC4100 Δ*murQ*::FRT | (10) | | |
| *B. subtilis* 168 | TrpC2; sequenced *B. subtilis* parental strain | Bacillus Genetic Stock Center | | |
| *B. subtilis* ∆*murQ* | *B. subtilis* 168 markerless *ybbI* (*murQ*) deletion mutant | this study | | |
| *B. subtilis* ∆*murQRP* | *B. subtilis* 168 markerless *ybbIHF* (*murQRP*) deletion mutant | this study | | |
| *B. subtilis* 168 pX | *B. subtilis* 168, *amyE*::pX; used as negative control (parental) strain, Cam^r^ | this study | | |
| *B. subtilis* 168 pX-*murQ* | *B. subtilis* 168, *amyE*::p*murQ*; xylose-inducible expression of *ybbI* (*murQ*), Cam^r^ | this study | | |
| *S. aureus* USA300 JE2 | USA300 LAC, lacking 3 plasmids for antibiotic resistance, parental strain | NARSA strain collection | | |
| *S. aureus* pRB474 | JE2 transformed with pRB474, Cam^r^ | this study | | |
| *S. aureus murQ*::*lox66-ermB-lox71* | JE2 *SAUSA300_0193*::*ermB* with lox66 and lox71, Erm^r^ | this study | | |
| *S. aureus* ∆*murQ* | JE2 markerless *SAUSA300_0193* (*murQ*) deletion mutant with lox72 | this study | | |
| *S. aureus* operon::*lox66-ermB-lox71* | *SAUSA300_0192- 0195* (*murQPR*) deletion mutant of JE2 with *ermB* resistance cassette, Erm^r^ | this study | | |
| *S. aureus* ∆*SAUSA300_0192-0195* | JE2 markerless *SAUSA300_0192* to *SAUSA300_0195* deletion mutant with lox72 | this study | | |
| *S. aureus* ∆*murQ* pRB474 | JE2 ∆*murQ* transformed with pRB474, Cam^r^ | this study | | |
| *S aureus* ∆*murQ* pRB474-*murQ* | JE2 ∆*murQ* transformed with pRB474-*murQ*, Cam^r^ | this study | | |
| *Streptomyces coelicolor* M145 | SCP1^-^, SCP2^-^, parental strain | (11) | | |
| *Streptomyces coelicolor* M145 ∆*murQ* | *SCO4307* deletion mutant of M145 | this study | | |

* Amp^r^ –ampicillin resistence; Apr^r^ –apramycin resistance; Cam^r^ –chloramphenicol resistence; Erm^r^ –erythromycin resistence; Tc^r^ –tetracycline resistence

**References**

1. **Geiger T, Francois P, Liebeke M, Fraunholz M, Goerke C, Krismer B, Schrenzel J, Lalk M, Wolz C.** 2012. The stringent response of *Staphylococcus aureus* and its impact on survival after phagocytosis through the induction of intracellular PSMs expression. PLoS Pathog **8:**e1003016.

2. **Leibig M, Krismer B, Kolb M, Friede A, Götz F, Bertram R.** 2008. Marker removal in staphylococci via Cre recombinase and different lox sites. Appl Environ Microbiol **74:**1316-1323.

3. **Brückner R.** 1992. A series of shuttle vectors for *Bacillus subtilis* and *Escherichia coli*. Gene **122:**187-192.

4. **Szurmant H, Mohan MA, Imus PM, Hoch JA.** 2007. YycH and YycI interact to regulate the essential YycFG two-component system in *Bacillus subtilis*. J Bacteriol **189:**3280-3289.

5. **Janes BK, Stibitz S.** 2006. Routine markerless gene replacement in *Bacillus anthracis*. Infect Immun **74:**1949-1953.

6. **Kim L, Mogk A, Schumann W.** 1996. A xylose-inducible *Bacillus subtilis* integration vector and its application. Gene **181:**71-76.

7. **Hanahan D.** 1983. Studies on transformation of *Escherichia coli* with plasmids. J Mol Biol **166:**557-580.

8. **Monk IR, Shah IM, Xu M, Tan MW, Foster TJ.** 2012. Transforming the untransformable: application of direct transformation to manipulate genetically *Staphylococcus aureus* and *Staphylococcus epidermidis*. MBio **3**.

9. **Casadaban MJ.** 1976. Transposition and fusion of the lac genes to selected promoters in *Escherichia coli* using bacteriophage lambda and Mu. J Mol Biol **104:**541-555.

10. **Jaeger T, Arsic M, Mayer C.** 2005. Scission of the lactyl ether bond of N-acetylmuramic acid by *Escherichia coli* "etherase". J Biol Chem **280:**30100-30106.

11. **Keijser BJ, van Wezel GP, Canters GW, Kieser T, Vijgenboom E.** 2000. The ram-dependence of *Streptomyces lividans* differentiation is bypassed by copper. J Mol Microbiol Biotechnol **2:**565-574.
