# Supplementary material for: Peptidoglycan Recycling in Gram-Positive Bacteria Is Crucial for Survival in Stationary Phase
Source: mBio. 2016 Oct 11;7(5):e00923-16. doi: 10.1128/mBio.00923-16 (PMC5061867; doi:10.1128/mBio.00923-16)
Supplement: Table S3 — Determination of the titers of viable S. aureus and B. subtilis wild-type (WT) parental and ΔmurQ cells grown in LB medium with or without MurNAc. S. aureus wild-type and ΔmurQ mutant cells were grown in LB medium in the absence or presence of 0.2% MurNAc. Viable cells were determined by counting CFU/ml (×108) at mid-exponential (4 h), transition (8 h), and stationary (24 h, 48 h, and 72 h) phase. B. subtilis wild-type and ΔmurQ cells were grown in LB medium in the absence or presence of 0.2% MurNAc to mid-exponential (4.5 h), transition (10 h), and stationary (24 h, 48 h, and 72 h) phase. Viable cell counts are presented as mean values ± SEM from three biological replicates. [file mbo005163019st3.docx]

**Table S3. Determination the titer of viable *S. aureus* and *B. subtilis* parental and ∆*murQ* cells grown in LB medium -/+ MurNAc.** *S. aureus* parental (WT) and ∆*murQ* mutant cells were grown in LB medium in the absence or presence of 0.2% MurNAc. Viable cells were determined by counting the colony forming units per ml (cfu/ml x10^8^) at mid exponential (4 h), transition (8 h) and stationary (24 h, 48 h and 72 h) phase. *B. subtilis* WT and ∆*murQ* cells were grown in LB medium in the absence or presence of 0.2% MurNAc to mid exponential (4.5 h); transition (10 h) and stationary (24 h, 48 h and 72 h) phase. Cfu/ml (x10^8^) were defined and presented as mean ± SEM from three biological replicates.
